# Protein evolution of Toll-like receptors 4, 5 and 7 within Galloanserae birds

**DOI:** 10.1186/s12711-014-0072-6

**Published:** 2014-11-12

**Authors:** Michal Vinkler, Hana Bainová, Josef Bryja

**Affiliations:** Department of Zoology, Faculty of Science, Charles University in Prague, Praha, Czech Republic; Institute of Vertebrate Biology, Academy of Sciences of the Czech Republic, v.v.i., Brno, Czech Republic

## Abstract

**Background:**

Toll-like receptors (TLR) are essential activators of the innate part of the vertebrate immune system. In this study, we analysed the interspecific variability of three TLR (bacterial-sensing TLR4 and TLR5 and viral-sensing TLR7) within the Galloanserae bird clade, investigated their phylogeny, assessed their structural conservation and estimated site-specific selection pressures.

**Results:**

Physiochemical properties varied according to the TLR analysed, mainly with regards to the surface electrostatic potential distribution. The predicted ligand-binding features (mainly in TLR4 and TLR5) differed between the avian proteins and their fish and mammalian counterparts, but also varied within the Galloanserae birds. We identified 20 positively selected sites in the three TLR, among which several are topologically close to ligand-binding sites reported for mammalian and fish TLR. We described 26, 28 and 25 evolutionarily non-conservative sites in TLR4, TLR5 and TLR7, respectively. Thirteen of these sites in TLR4, and ten in TLR5 were located in functionally relevant regions. The variability appears to be functionally more conserved for viral-sensing TLR7 than for the bacterial-sensing TLR. Amino-acid positions 268, 270, 343, 383, 444 and 471 in TLR4 and 180, 183, 209, 216, 264, 342 and 379 in TLR5 are key candidates for further functional research.

**Conclusions:**

Host-pathogen co-evolution has a major effect on the features of host immune receptors. Our results suggest that avian and mammalian TLR may be differentially adapted to pathogen-derived ligand recognition. We have detected signatures of positive selection even within the Galloanserae lineage. To our knowledge, this is the first study to depict evolutionary pressures on Galloanserae TLR and to estimate the validity of current knowledge on TLR function (based on mammalian and chicken models) for non-model species of this clade.

**Electronic supplementary material:**

The online version of this article (doi:10.1186/s12711-014-0072-6) contains supplementary material, which is available to authorized users.

## Background

Toll-like receptors (TLR) are part of the pattern recognition receptor machinery and play a key role in initial pathogen recognition in vertebrates [1]. Since TLR are responsible for the recognition of microbe-associated molecular patterns that are present on pathogens [2], the efficiency of innate immunity in vertebrates is conditioned by their optimal functioning. Substantial variability has been described for TLR, both at the interspecific and intraspecific level [3]. Variability in the structure and binding features of TLR could significantly influence host resistance to diseases and vulnerability to autoimmune damage. TLR evolution has been intensively studied in vertebrates in general [4–6] and within the mammalian clade in particular [7–10]. In birds, although the number of studies on the evolution of TLR has steadily increased [11–13], there is still a very limited understanding of the functional significance of the putatively adaptive variability observed.

There are ten *TLR* genes in birds [5,11,14], including those encoding predominantly bacterial-sensing TLR, such as TLR4 and TLR5, and viral-sensing TLR, such as TLR7. Although the TLR and their corresponding genes have been well characterised in the domestic chicken (*Gallus gallus domesticus* [15–21]), our knowledge on TLR of other avian species remains rather fragmentary [8,22–31]. Based mainly on data for the domestic chicken, we assume that the ligands recognized by avian TLR are similar to their mammalian counterparts [14], i.e. TLR4 binds lipopolysaccharide (LPS) [32], TLR5 binds flagellin [33] and TLR7 binds viral single-stranded RNA and synthetic antivirals [19,34]. The structure of bacterial ligands, such as LPS or flagellin, varies between species [35–37], which may exert selective pressure on TLR and lead to host-pathogen co-evolution of these molecules [3]. Previously, it has been shown that TLR-ligand binding is, in many aspects, species-specific and that TLR protein variation may have a functional significance [32,33,38–41] that could affect resistance to pathogen infections [16,42].

The function of the receptor is determined by its tertiary structure and surface features that confer specificity for ligands. TLR are type I integral membrane glycoproteins that are characterized by an extracellular ligand-binding domain (ECD) and a cytoplasmic signalling Toll/IL-1R homology (TIR) domain. Three-dimensional (3D) molecular structures of the ECD [43–50] and TIR domains [51,52] have been described for several human and mouse TLR. In several other cases, the ECD structures are predicted [53–58]. Most recently, the TLR5 ECD structure has been resolved in zebrafish (*Danio rerio* [59]). These studies have identified TLR ligand-binding sites. All these protein structures, however, represent mammalian or fish TLR only, no avian TLR have been characterised or even predicted in 3D as yet.

In this study, we provide a comprehensive view on the evolution of TLR in Galloanserae birds. Galloanserae is an ancient clade of avian species that includes the orders Galliformes and Anseriformes which are largely separated from all other modern birds of the Neoaves lineage [60]. Currently, this is the most extensively studied avian taxon regarding TLR immunogenetics. Based on published data, we investigated the sequence and structural variability of TLR4, TLR5 and TLR7 within this bird clade. These three TLR were chosen as representatives of the bacterial-sensing and viral-sensing TLR based on published sequence data. Since comparison of 3D structures and protein surface features may reveal biologically interesting similarities not detectable by sequence analysis [61], the 3D tertiary structure of these three proteins was modelled. We then carried out a structural comparison of functionally important regions, a comparison of surface electrostatic potentials and four independent analyses of positive selection. Superposition of the 3D structures allowed us, for the first time, to conduct a phenetic (not only phylogenetic) analysis of avian TLR evolution.

## Methods

### Input sequences

Coding DNA sequences (CDS) for the selected TLR were downloaded from the National Centre for Biotechnology Information (NCBI) GenBank for all Galloanserae species currently available in full length (n = 13) and for humans and mouse. GenBank ID are given in Additional file 1: Table S1. CDS translation was performed using BioEdit Sequence Alignment Editor (Tom Hall, Ibis Biosciences, Carlsbad, California, USA) and protein sequences for each gene were aligned using ClustalW multiple-sequence alignment software. Alignment of nucleotide codons and their corresponding protein sequences was undertaken using the PAL2NAL webtool [62].

### Structural analysis

To predict the distribution of structural domains in the proteins, we applied SMART [63]. Since the analysis revealed differences between species in the number and position of leucine-rich repeats (LRR) we tested LRR distribution by an independent approach using the LRRfinder, with upper and lower boundaries fixed at 95% and 80%, respectively [64]. Because the results of these two approaches differed slightly, SMART predictions were only used to identify N-terminal LRR (LRRNT) and C-terminal LRR (LRRCT) motifs which in most cases were not identified by LRRfinder, while other LRR were identified based on LRRfinder predictions. Molecular weight and charge at pH = 7 were also calculated for each predicted protein. Presence of a transmembrane (TM) region in each protein was checked for on the DAS-TMfilter server [65]. When the TIR domain was not detected by SMART, we used PFAM comparison [63] to check for its presence. Signal peptides were identified using SignalP 4.0 [66]. Finally, secondary and 3D tertiary structures of the three TLR were predicted by applying a comparative modelling approach (see e.g. [56,57] or [55]) using I-TASSER [67], which is currently the leading protein structure prediction server (see [68]) and http://predictioncenter.org/casp10/)). The I-TASSER server uses a hierarchical protein-structure modelling approach based on secondary-structure enhanced profile-profile threading alignment and iterative implementation of the threading assembly refinement program [68]. For the prediction of secondary structures, we used the whole CDS, whereas ECD and TIR domains were modelled separately in order to compare tertiary structures. Since it has been shown that the sequence for TLR7 up to LRR14 is cleaved in the endoplasmic reticulum [69], we modelled the sequence starting at amino-acid 417 only. Models with the highest C-scores and conformation similarities to other modelled structures were used for further analysis. In these models, we excluded regions with limited structural stability (i.e. regions with high modelling errors: signal peptides and regions following LRRCT). Modelling errors in the regions of interest were estimated using ModFOLD [70]; all models had high levels of confidence with P-values less than 0.002 and Global model quality scores greater than 0.37. Although the accuracy of our models may still have been limited, the aim of this study was not to describe the proteins’ tertiary structures precisely but to assess average structural similarity between the receptors. The error estimates obtained indicate that the models constructed represent reasonably reliable inputs for further phenetic analysis. Images of the predicted protein 3D structures were visualised using PyMOL software v. 1.5 (http://pymol.org/). Protein electrostatic potentials were calculated using PDB2PQR v. 1.9.0 [71] (PDB2PQR Server, http://nbcr-222.ucsd.edu/pdb2pqr_1.9.0/) based on the PARSE force-field and electrostatic calculation in the APBS web solver [72] (http://www.poissonboltzmann.org/). Surface charge distribution was visualised using Jmol v. 12.2 (http://jmol.sourceforge.net/).

### Phylogenetic and phenetic analysis

Alignments of *TLR4*, *TLR5* and *TLR7* CDS were used for phylogenetic analysis using a maximum likelihood (ML) method. As outgroups, we used orthologous human and mouse sequences. FindModel (http://www.hiv.lanl.gov/content/sequence/findmodel/findmodel.html) was used to evaluate the fit of 28 nested nucleotide substitution models to the data, the best model for each alignment being selected on the basis of the Akaike information criterion. ML analyses were performed using PHYML [73], with the NNI algorithm and BIONJ distance-based tree as the starting tree. Bootstrap analysis (with 1000 replicates) was performed to estimate the robustness of internal nodes. The results were visualised in FigTree v. 1.3.1 (http://tree.bio.ed.ac.uk/software/figtree/). A consensus phylogenetic tree including all investigated species (see Additional file 2: Figure S1) was constructed using the avian phylogenetic tool available at http://birdtree.org/ [74]. We used the Hackett backbone [60] as the source tree with 1000 randomly generated trees. The maximum clade credibility tree was produced using the TreeAnnotator v. 1.8.0 tool in BEAST v. 1.8.0 software [75]. Phenetic similarity analysis of the predicted protein secondary and tertiary structures was performed to detect conserved structures in avian TLR. Secondary structures were compared based on alignments obtained using the EMBOSS Needle pairwise alignment tool (http://www.ebi.ac.uk/Tools/psa/emboss_needle/), using chicken (*Gallus gallus*) GaGaTLR sequences as references. To predict 3D structures, we used the adjusted I-TASSER pdb models for structural superposition in the DALI pairwise comparison tool [61]. The pair-wise root mean square deviations (RMSD) metric was used to compare protein structures [76,77] and to construct distance matrices that subsequently served as matrices of Euclidean distances in cluster analysis using an unweighted pair group method with arithmetic mean (UPGMA) method in STATISTICA v. 6.0 (StatSoft, Inc., Tulsa, OK, USA; [78]; for a similar approach see [10]).

### Selection analysis

Before testing for selection, all codons containing gaps in any species in the alignment were removed (this applied to only six codons in TLR5; throughout the text, codon positions are numbered according to the chicken GaGaTLR5 sequence (see Additional file 1: Table S1). We used two methods to test for positive selection on individual residues at the interspecific level within the Galloanserae clade, i.e. (1) the hierarchical Bayes (Bayes Empirical Bayes, BEB) approach with implemented Markov chain Monte Carlo routine - PAML (Phylogenetic Analysis by Maximum Likelihood [79]); and (2) FUBAR (A Fast, Unconstrained Bayesian AppRoximation for inferring selection [80]). For PAML (v. 4.6), we used codon-based substitution models (codeml) to identify amino acid sites under positive selection in the CDS comparing the neutral M8a (beta&ω = 1) model with the alternative M8 (beta&ω) model. The likelihood ratio test (LRT) for the comparison of two nested models was calculated using chi-square approximation: Chi^2^ = 2 × (lnLM8 – lnLM8a), where LM8 and LM8a are likelihood values. The degrees of freedom (df) were defined as the difference in the number of parameters in the models used (see Additional file 1: Table S6). If the LRT was less than 0.05, positive selection was considered significant. The BEB approach [81] was used to determine site-specific posterior probabilities of positive selection (≥0.9). FUBAR analysis was performed on the Datamonkey server (http://www.datamonkey.org/, [82]) using a default significance level of posterior probability set at 0.9. In this study, we applied the FUBAR algorithm because it is more robust and much faster than selection analysis based on random effect likelihood (REL methods [80]).

We tested the degree of dissimilarity of amino acid substitutions according to their physiochemical properties using the new PRIME (PRoperty Informed Model of Evolution) tool available on the Datamonkey server [82]. We used the set of five composite physiochemical properties proposed by Atchley et al. [83], i.e. polarity index, secondary structure factor, volume, refractivity/heat capacity and charge/iso-electric point. A change in these properties was considered significant if the posterior probability was greater than 0.9. The evolutionary conservation of amino acid positions was predicted using the ConSurf tool [84], with the assumption that positively selected residues (functionally important for pathogen binding) were the least conserved. For all ConSurf analyses, we used GaGaTLR protein 3D models (obtained as described above) and the LG substitution matrix [85]. A phylogenetic tree of the three *TLR* genes studied including all investigated species in the PAML and ConSurf analyses was constructed as described above (see Additional file 2: Figure S1).

## Results and discussion

We were able to verify the homology of all sequences examined (see Additional file 2: Figure S2) and subsequently to assess amino acid identity and similarity (see Additional file 1: Table S2). The phylogeny of the *TLR* genes was consistent with known phylogeny for the Galloanserae clade [86] (see Additional file 2: Figures S1 and S2). While the size of both TLR4 and TLR7 was uniform (843 aa and 1047 aa, respectively), TLR5 varied in length between 859 aa and 862 aa (see Additional file 1: Table S3 and Additional file 3: Section S1). The orthologues showed little variation in molecular weights, although some differed markedly in their charge at pH = 7 (see Additional file 1: Table S3). Since localised charge variability can influence protein conformation and domain composition and produce variation in ligand-binding features, we examined charge and structural variation in more detail.

### Protein structure evolution

TLR proteins are likely to be involved in host-parasite co-evolution, and thus shaped by parasite-mediated natural selection [3]. Interspecific differences in TLR protein structure, therefore, may exhibit imprints of structural evolutionary convergence due to selection. In this study, for the first time in birds, we were able to model secondary and tertiary structures of TLR4, TLR5 and TLR7 for all Galloanserae species with currently known CDS. Secondary structures for the proteins predicted by I-TASSER revealed low levels of interspecific structural variability within individual TLR (see Additional file 2: Figure S3). Although two regions of potential functional interest in TLR4 were polymorphic (see Additional file 3: Section S2), the most important structural motifs in all three receptors were conserved in all species analysed, resulting in more than 90% interspecific identity in secondary structure distribution (see Additional file 1: Table S4). As a result, 3D extracellular domain models invariably had a horseshoe-like shape, in which the concave surface comprised β-sheets and the convex surface parallel loops and short helices. The TIR domain (which was modelled separately) had a globular shape. A phenetic analysis of RMSD distances obtained by superposition of the modelled structures revealed that structural variability in the TIR domain of all three receptors was lower than ECD variability (see Additional file 2: Figures S4d, S4e and S4f). This may be a result of higher conservation in the TIR domain when compared to ECD [5], although domain size may also have played a role as RMSD tends to increase with protein size. Avian TLR4 ECD showed a stronger structural resemblance to human TLR4 than mouse TLR4 (see Additional file 2: Figure S4a), which suggests that avian TLR4 may exhibit similar binding features to human TLR4. At present, however, this must remain a hypothesis since current experimental data provide no support [32]. As the RMSD of our superposed models were below 2.5 Å (i.e. deviation between models was lower than the accuracy of individual models), we conclude that TLR4 and TLR5 ECD structural variability within the Galloanserae is generally low and probably unimportant (see Additional file 2: Figures S4a and S4b). Of more interest is the ECD of TLR7, with the phenogram indicating that Anseriform TLR7 exhibits a close structural relationship to human TLR7, while Galliform TLR7 clusters with murine TLR7 (see Additional file 2: Figure S4c). Despite this, the RMSD were too low to indicate any meaningful structural variation. The low TLR structural variability observed in this study is consistent with recent findings for rodents [10].

### Surface electrostatic potential

**Figure 1:**
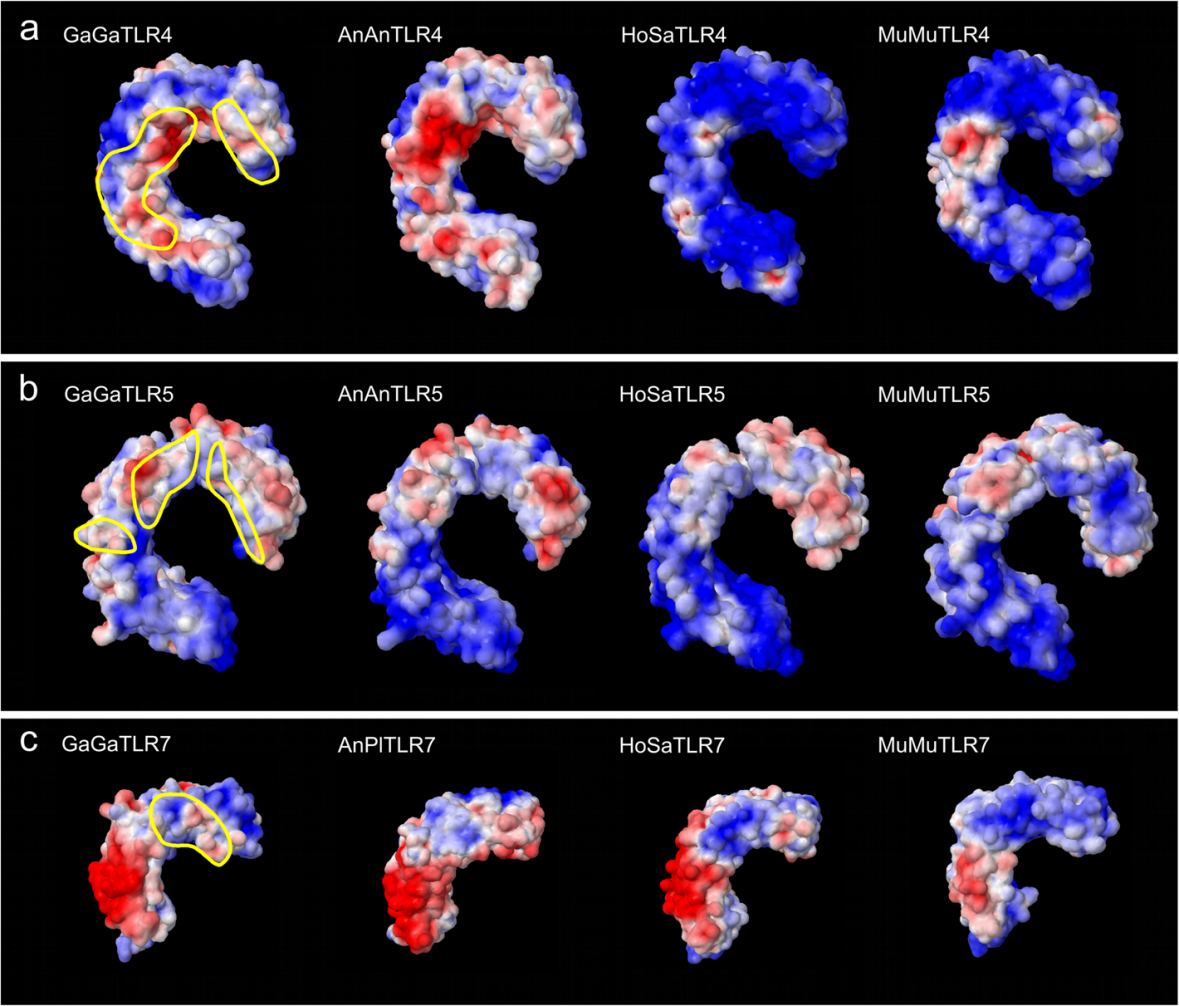
**Differences in ECD surface electrostatic potentials between birds and mammals (for a full comparison see Additional file** **2** **: Figure S5). (a)** TLR4, **(b)** TLR5, **(c)** TLR7; positive surface charge is highlighted in red, negative charge in blue; the predicted functional sites in ECD are outlined in yellow in the GaGaTLR models; GaGa = chicken *Gallus gallus*, AnAn = goose *Anser anser*, AnPl = duck *Anas platyrhynchos*, HoSa = human *Homo sapiens*, MuMu = mouse *Mus musculus*.

After showing that the protein tertiary structures were highly conserved, we ascertained interspecific differences in surface features by modelling electrostatic potential distribution on the TLR protein surfaces. While TIR domain surface charge distribution remained relatively conserved, we observed high variability in TLR ECD (see Additional file 2: Figure S5). Anseriform TLR ECD differed from ECD of their Galliform counterparts. Species-specific differences were observed even between individual species within the Galliformes. Avian TLR4 ECD surface-charge distribution was clearly distinguishable from that of mammalian (murine and human) TLR4, although surface charge for the predicted avian ligand-binding region [48,49], in particular, was clearly closer to that of murine TLR4 than human TLR4 (Figure 1a). This is consistent with a previous observation indicating that GaGaTLR4 LPS-binding specificity shows greater similarity to that of murine TLR4 than human TLR4 [32]. Variability was much lower in TLR5 ECD; although, once again, avian TLR5 charge distribution at the flagellin-binding site predicted for mammals [54] was closer to that for murine TLR5 than human TLR5 (Figure 1b). As for TLR4, these results are in concordance with the results of previous functional assays [33]. In contrast, for both TLR7 (Figure 1c) and the flagellin-binding interface-A region identified in zebrafish [59], electrostatic potential distribution at the predicted ligand-binding interface [56] resembled that of human TLR more than murine TLR.

### Changes in physiochemical features of predicted ligand-binding residues

To further explore potential interspecific variability in the ligand-binding properties of avian TLR proteins, we compared basic chemical features of residues known or predicted to be functionally important in other vertebrate TLR [38,48,49,54,56,59]. These included residues directly involved in ligand binding, TLR homodimerization and MD-2 heterodimerization. At most of the predicted functional sites in avian TLR, similar chemical properties were preserved as in mammalian TLR (see Additional file 1: Table S5). Such conservation was particularly visible in TLR7, which suggests that most receptor-ligand interactions in TLR7 are highly conserved (see Additional file 3: Section S3). In TLR5, we identified substantial residue differences at the binding region previously described in zebrafish [59] as separating fish from amniotes (see Additional file 1: Table S5). Primary binding interfaces A and B, as well as both dimerization interfaces, appear to be only modestly conserved, indicating that flagellin binding probably differs in fish and amniotes. This is further supported by the fact that avian TLR5 sites generally retain their physiochemical properties at those binding residues predicted for mammals. Intriguingly, we identified potentially important changes in amino acid properties at several TLR4 functional sites, which suggests variability in LPS binding and MD-2 dimerization (see Additional file 1: Table S5). In a number of cases, residue changes precluded the existence of charge (positions 268 and 397) or hydrophobic interactions (positions 449 and 472) known for human TLR4 [49,87]. Interestingly, all avian species possess arginine at position 393; in this respect, avian TLR4 is identical to equine TLR4 [38] but distinct from human TLR4. This suggests that lipid IVa in birds serves as an agonist triggering a TLR4-MD-2-mediated immune response, just as it does in horses [38]. Galliformes also display systematic differences from Anseriformes at several positions (see Additional file 3: Section S3). Thus, our results suggest that the ligand-binding features of avian TLR differ not only from mammalian TLR but also between the various avian taxa. This is in concordance with previously reported experimental results [32,33].

### Analysis of positive selection

**Figure 2:**
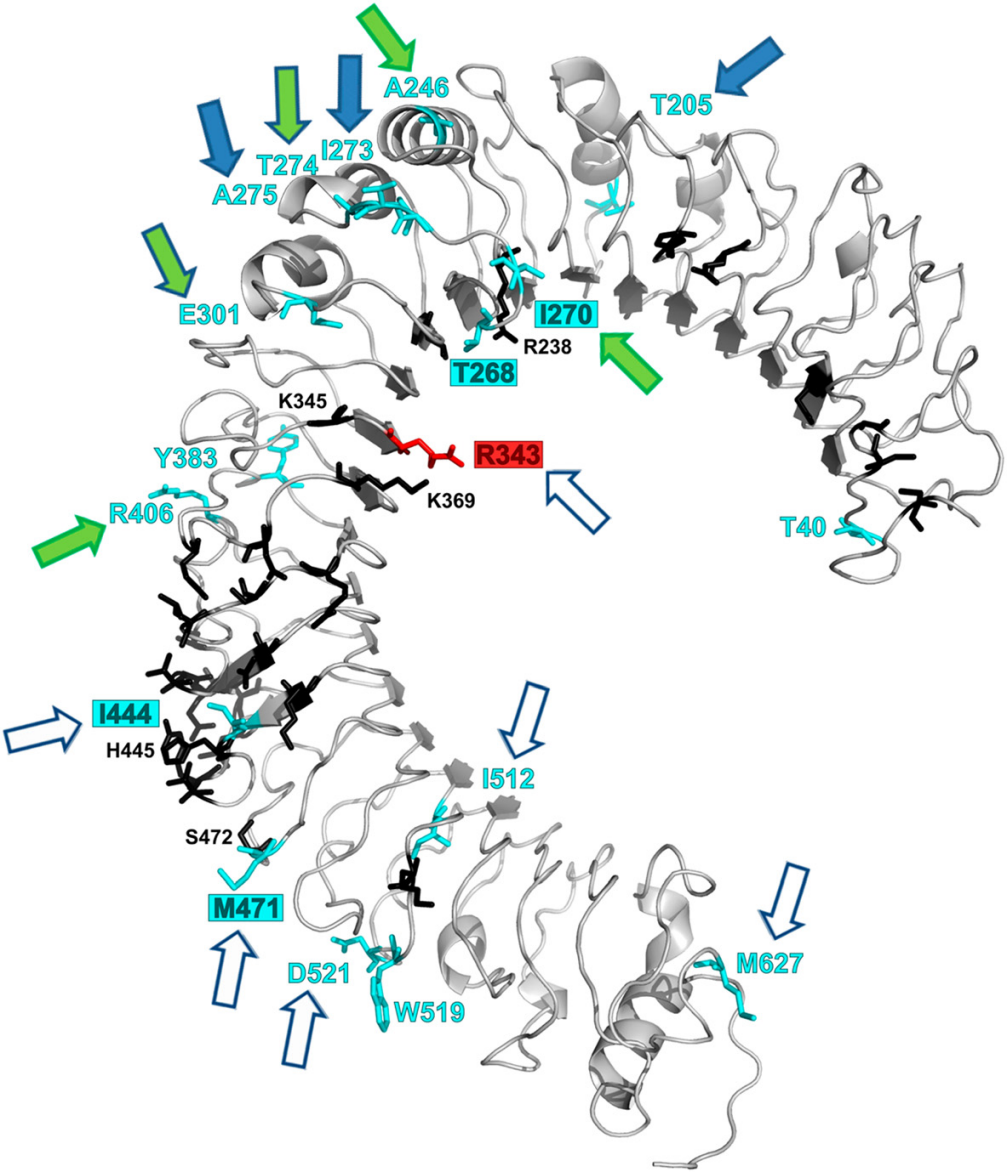
**Projection of detected positively selected sites (PAML/FUBAR/PRIME) and evolutionarily non-conservative sites (ConSurf) onto a 3D protein model of chicken GaGaTLR4 ECD.** Positively selected sites are highlighted in red, evolutionarily non-conservative sites in blue and functionally important sites known from mammalian studies [38,48–50,87] in black (only positions lying in close proximity to the selected or non-conservative sites are numbered); GaGaTLR residue numbering has been adopted; selected sites or non-conservative residues located in close proximity to any of the predicted functional sites are indicated by a filled red or blue rectangle, respectively; arrows indicate positively selected sites concurrent with those identified in previous studies (filled green = avian, filled blue = mammalian, filled green with a blue border = avian and mammalian, open blue = site in neighbourhood); position 268 has been identified in mice and humans as a site directly responsible for MD-2 dimerization and LPS binding.

**Figure 3:**
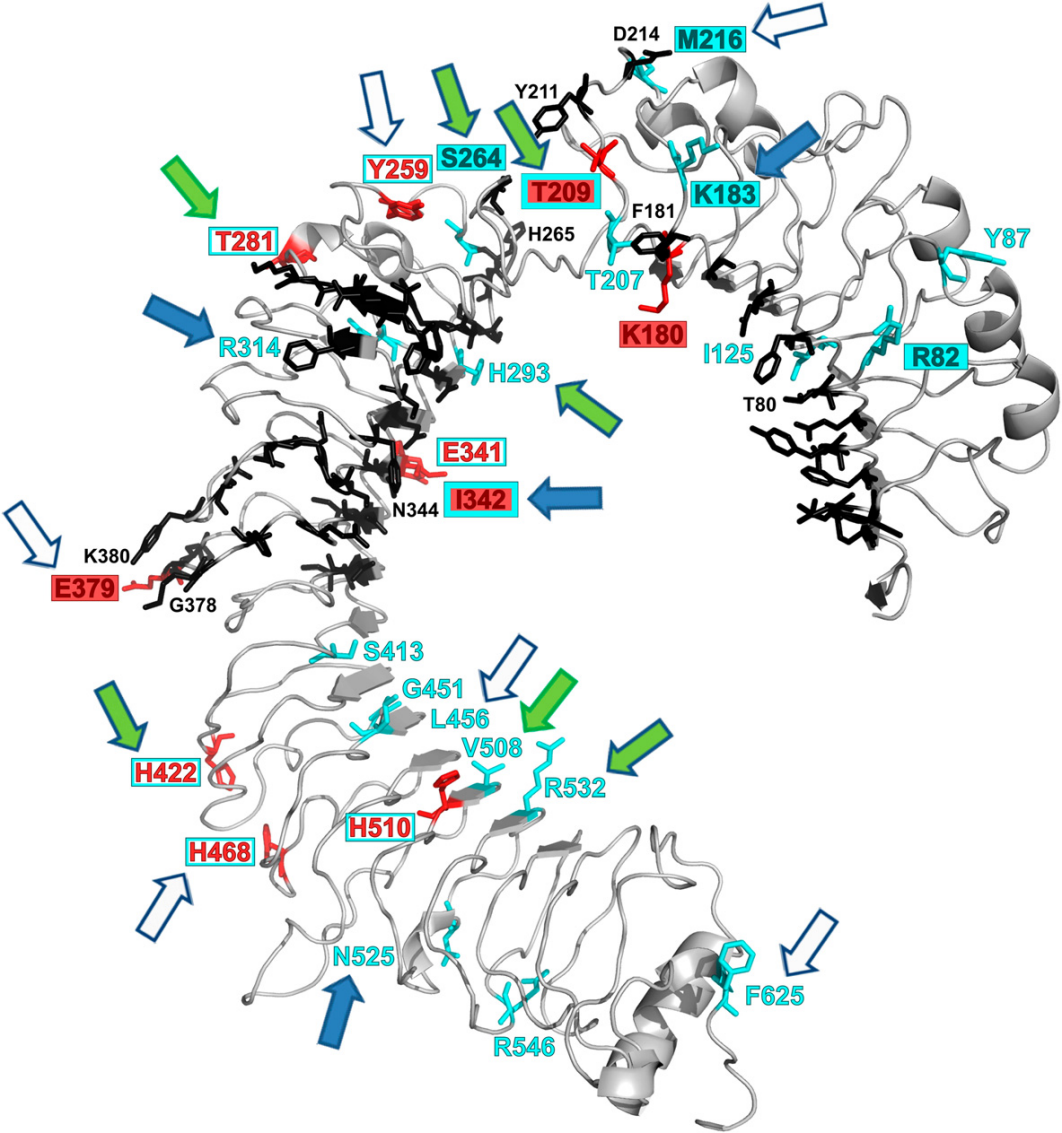
**Projection of detected positively selected sites (PAML/FUBAR/PRIME) and evolutionarily non-conservative sites (ConSurf) onto a 3D protein model of chicken GaGaTLR5 ECD.** Positively selected sites are highlighted in red, evolutionarily non-conservative sites in blue and functionally important sites known from fish [59], and predicted for mammals [54], are in black (only positions lying in close proximity to the selected or non-conservative sites are numbered); GaGaTLR residue numbering has been adopted; selected sites or non-conservative residues located in close proximity to any of the predicted functional sites are indicated by a filled red or blue rectangle, respectively; overlap of the identified selected positions with non-conservative residues is indicated by a red number in an open blue rectangle; arrows indicate positively selected sites concurrent with those identified in previous studies (filled green = avian, filled blue = mammalian, filled green with blue border = avian and mammalian, open blue = site in neighbourhood); position 209 has been identified as both positively selected and non-conservative; in addition, this residuum (209) is also known as a flagellin-binding site in fish, as are residues 183 and 379; position 342 is both positively-selected and non-conservative and lies in close proximity to residuum 344 which has been recognised as a possible flagellin-binding site in mammals.

**Figure 4:**
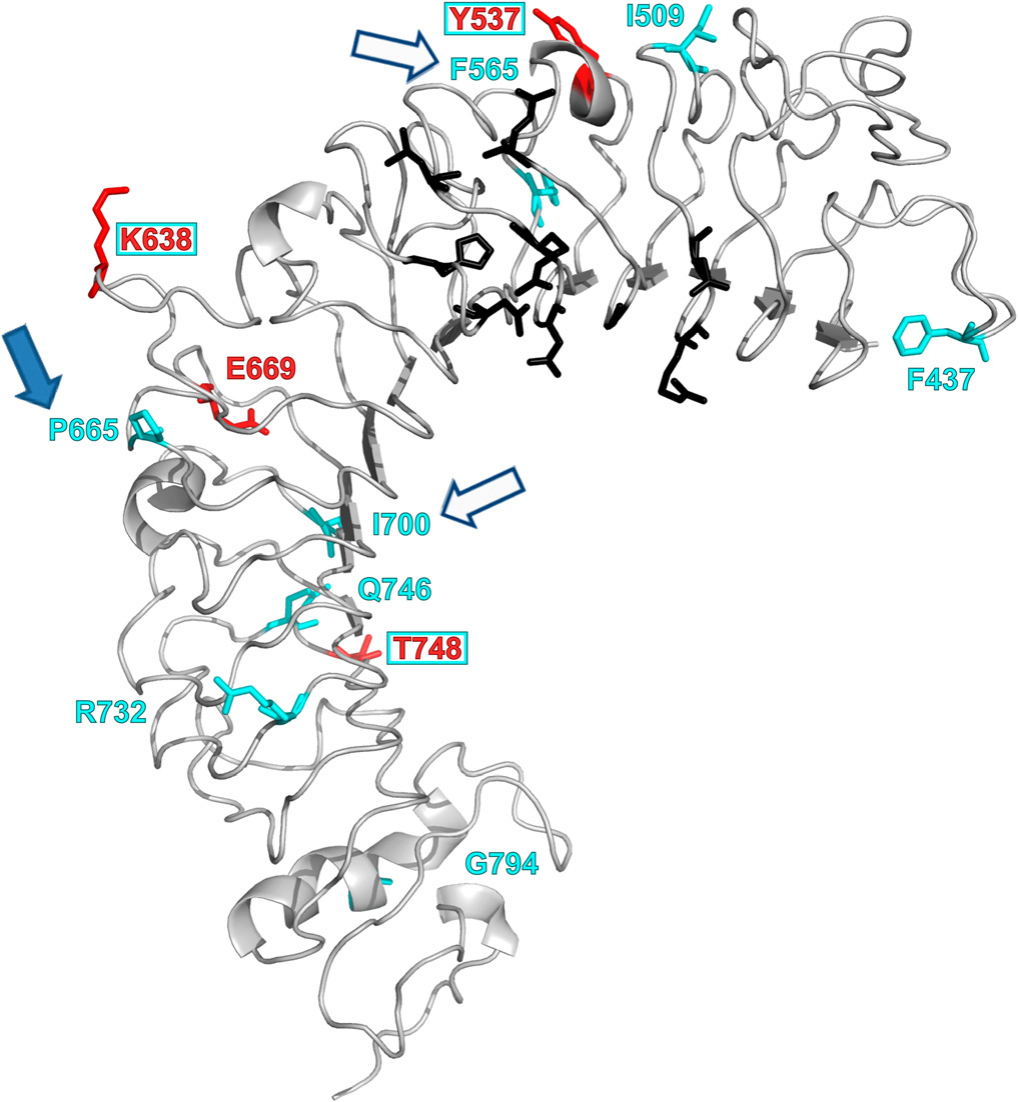
**Projection of detected positively selected sites (PAML/FUBAR/PRIME) and evolutionarily non-conservative sites (ConSurf) onto a 3D protein model of chicken GaGaTLR7 ECD.** Positively selected sites are highlighted in red, evolutionarily non-conservative sites in blue and functionally important sites predicted for mammals [56] are in black; GaGaTLR residue numbering has been adopted; overlap of the identified selected positions with non-conservative residues is indicated by a red number in an open blue rectangle; arrows indicate positively selected sites concurrent with those identified in previous studies (filled blue = mammalian, open blue = site in neighbourhood).

Chicken *TLR* genes show remarkable differences in the level of sequence polymorphism, most likely as a result of distinct positive and negative selection [6,20,31]. This is further supported by analysis of partial *TLR* CDS in previous studies on avian species [12,13]. In this study, we investigated signatures for positive, diversifying selection acting on individual positions in TLR4, TLR5 and TLR7 within the Galloanserae lineage. We identified one positively selected site in TLR4, 11 sites in TLR5 and eight sites in TLR7, using three different methods (Figures 2, 3 and 4). Two of the approaches used were based on site-by-site synonymous and non-synonymous rate ratios (PAML and FUBAR), which detected seven positively selected sites in both TLR5 and TLR7 but no positively selected sites in TLR4 (see Additional file 1: Table S7). The lack of evidence for positive selection in TLR4 is probably due to the low power of analysis because of a limited number of distantly-related species (all species of the genus *Gallus* are genetically closely-related, and thus display similar genetic sequences); hence, in this case, the lack of evidence should be treated as a probable methodological artefact. The third method which used a physiochemical comparative approach (PRIME), detected positive selection in all three proteins, with one positively selected site in TLR4, five sites in TLR5 and two sites in TLR7 (see Additional file 1: Table S8). Of the 20 positively selected sites detected in total, only one was located in the transmembrane domain, the others being located in ECD. No positive selection signatures were detected in the intracellular TIR domain, which suggests that positive selection acts mainly on ligand-binding regions. This confirms the results of similar analyses undertaken on mammals [7,9,10,88,89] and amniotes [22]. Projection of positively selected and functionally important sites onto 3D protein models of GaGaTLR revealed changes at six positions (TLR4: 343; TLR5: 180, 209, 342, 379; TLR7: 26) that could directly influence receptor expression or function (Figures 2, 3 and 4, see Additional file 1: Tables S7 and S8 and Additional file 3: Section S4). As in other birds [12,13], Galloanserae TLR5 displayed a relatively high accumulation of codons that exhibit positive selection. This is in concordance with the results of Wlasiuk et al. [7] reported for primates. On the other hand, TLR7 has previously been shown to evolve mainly under purifying selection in birds; with no or only limited positively selected sites in the predicted ligand-binding region [9,10,12,13]. In this study, we were able to detect some positively selected sites (Figure 4; see Additional file 1: Tables S7 and S8); however, given their locations (except for position 26), these sites appear to have only limited functional importance and their impact remains unclear.

While the vast majority of residues in TLR were evolutionarily highly conservative, consistent with purifying selection on TLR [6,10,13], ConSurf analysis revealed 26 reliable evolutionarily non-conservative sites in TLR4, 28 in TLR5 and 25 in TLR7 (Figures 2, 3 and 4 and (see Additional file 1: Table S9 for detailed information). These may also represent positively selected sites since functionally important positively-selected residues are also predicted to be the least conserved. This is supported by the location of six of the non-conservative sites in the functionally important TLR4 signal peptide, four in the predicted TLR4-MD-2-binding regions [48,49], three in the LPS-binding regions [49,50], two in the TLR4-TLR4-dimerization regions [49] and one in the trans-membrane region (see Additional file 3: Section S4). One of the non-conservative positions identified in our Galloanserae dataset (383) has previously been identified as a possible single nucleotide polymorphism (SNP) responsible for *Salmonella* resistance in the domestic chicken [16]. In TLR5, one non-conservative site was situated in the signal peptide, five in the flagellin-binding residues identified in fish [59], two in flagellin-binding residues predicted in mammals [54] and two in the trans-membrane region. Most of the non-conservative sites in TLR7 were situated in the excised region [69] (and hence likely to be functionally unimportant), with just one in the potentially relevant signal peptide and one in the TIR domain.

Intriguingly, both the positively-selected and non-conservative sites identified in this study partly match the positively selected sites detected in previous studies in other vertebrate taxa [7,9,10,12,13,22,90,91] (Figures 2, 3 and 4, Additional file 1: Table S10 and Additional file 3: Section S4). In TLR4 and TLR5, 15 and 20 candidate sites, respectively, were either precisely the same or lay in close proximity (up to 2 aa) to a site identified in previous studies. In contrast, only seven sites were close to, or the same as, previously detected sites in TLR7. Most importantly, there was consensus for positive selection on site 270 in the predicted MD-2-binding region in TLR4 in birds, and on sites 183, 209, 264 and 342 in TLR5 both in birds and mammals, all of which are predicted flagellin binding sites or sites in close proximity to these binding sites (see Additional file 3: Section S4). No consensus site was located in the predicted ligand-binding region in TLR7. Independent concordance between the results of selection analysis in several studies that cover different taxa, and topological agreement between the identified sites and predicted functionally important regions, strongly support the proposed importance of diversifying selection or positive selection in the evolution of these residues. In human TLR4, for example, it has been demonstrated that even SNP located far from MD-2-dimerization or LPS-binding sites can modify responsiveness to LPS [92] or influence the binding of other ligands. Thus, other consensus selected sites identified in TLR4 (205, 246, 273–275, 301 and 406, Figure 2) and TLR5 (281, 293, 314, 422, 508, 525 and 532, Figure 3) may play an important role in the evolution of TLR-ligand binding in birds (see Additional file 1: Table S10). However, the potentially important consensus sites identified in TLR7, are located mainly in the excised region [69] (residues at positions 39, 99 and 383) and do not appear to influence receptor function. The only exception to this is site 665 which lies, however, outside the predicted ligand-binding region. Given the generally low concordance in predicted positively-selected sites between studies, we suggest that many of the sites reported for TLR7 represent false-positive predictions resulting from the method applied (most sites were identified by REL or FEL; see [80] for discussion).

In concordance with previous studies, we found stronger signatures of positive selection acting on the ligand-binding regions in TLR4 [9] and TLR5 [7] than in TLR7 [12]. There are several possible explanations for this. First, bacterial-sensing TLR such as TLR4 and TLR5 recognise structurally variable ligands [35–37], while viral-sensing TLR7 detects structurally invariant RNA molecules regardless of their precise sequence [93]. Hence, TLR7 is likely to have evolved mainly under purifying selection, while TLR4 and TLR5 evolved mainly under diversifying selection. Furthermore, since TLR4 is capable of recognising several unrelated ligands [3], it is also possible that this receptor evolved more rapidly. Second, there appears to be greater redundancy in bacterial recognition than viral recognition. In TLR5, for example, several studies have proposed relaxed purifying selection [7,9], while nonsense stop-codons have been described in both birds [94] and mammals [7]. This may be a result of the presence of other flagellin receptors which may compensate for malfunction of TLR5 (discussed in [94]). Finally, the limited number of positively selected sites detected in the TLR7 ligand-binding region may result from the limited knowledge about its precise location. While the precise mechanism of ligand-binding has been described for both TLR4 [48–50] and TLR5 [59], only crude predictions are available for TLR7 [56]. Although all these explanations may be relevant to some extent, the relatively low concordance between results of multiple studies involving TLR7 (see Additional file 1: Table S10) tends to support the view that there is, indeed, only weak positive selection acting on TLR7.

## Conclusion

Interspecific comparisons within the Galloanserae clade revealed relatively high sequence variability in all three TLR investigated. Such variation has been shown to influence the physiochemical properties of proteins. Despite high tertiary-structure conservation, evolutionary changes were manifested by alterations to protein surface characteristics, such as changes in electrostatic potential distribution. Importantly, not only does surface charge in Galloanserae birds differ distinctly from that of mammals, to some extent there are also distinct differences observed within the clade. These variations most likely affect receptor binding features, a theory that is consistent with the idea of a host-pathogen evolutionary arms race [95] in which any adaptation enabling a pathogen to escape host immunity leads inevitably to a counter-adaptation in host receptors that again enables pathogen detection. Co-evolution of this kind has previously been described in both human TLR4 and TLR4 of the bacterium *Pseudomonas aeruginosa* [39]. In fact, there are now numerous examples of individual host TLR adaptations to pathogens known (summarised, for example, in [3]). What we presently lack, however, is a functional understanding of evolutionarily-tested beneficial innovations present at the interspecific level in vertebrates. In this study, selection analysis identified a number of positively selected sites, mostly in the ligand-binding ECD. As in previous studies, we observed stronger positive selection acting on the ligand-binding regions of TLR4 [9] and TLR5 [7] compared to TLR7 [12]. Sites subjected to selection in TLR4 and TLR5 were frequently located either precisely in, or in close topological proximity to, ligand-binding sites known or predicted in mammalian or fish TLR. This suggests that, although avian TLR may be differentially adapted to pathogen-derived ligand recognition compared to that of other vertebrate species, identical regions are responsible for ligand binding. We suggest that future investigations in this field should focus on functional testing of evolutionarily relevant substitutions detected by selection analyses. Based on the evidence summarised above, we propose several sites for more detailed investigation. In particular, sites 270, 343, 444 and 471 in TLR4 and sites 183, 209, 216, 264, 342 and 379 in TLR5 represent key candidates for further research on the functional significance of selection acting on TLR in birds. We suggest that site 268 (and possibly also site 383) in TLR4 and site 180 in TLR5 may be of particular evolutionary importance in Galloanserae birds since no selection on these sites has previously been observed in either mammals or other birds. Furthermore, while we do not know the functional importance of the sites, concordance between selection analyses in both birds and mammals suggests that special attention should be paid to positions 244–246, 273–275, 301 in TLR4 and 293, 294, 314, 342, 422, 525, 532, 533 in TLR5.

Taken together, our results have identified variability in Galloanserae birds that very likely results from pathogen-mediated evolution of species-specific TLR binding features. To our knowledge, this is the first study to depict evolutionary pressures on Galloanserae TLR and to estimate the validity of current knowledge on TLR function (based on mammalian and chicken models) for non-model species of this clade. Functional testing of the importance of individual sites (such as that performed by Walsh et al. [38] in mammals) should provide novel understanding of evolutionary mechanisms increasing resistance to pathogens in avian species. Any knowledge gained would be of great practical relevance, with applications in animal breeding for increased resistance to diseases.

## Additional files

Additional file 1: Table S1. Specification of sequences obtained from NCBI GenBank. Detailed information on NCBI GenBank sequences analysed in this study. **Table S2.** Homology of GaGaTLRs with other Galloanserae TLR molecules. Nucleotide and amino acid identity and similarity calculated in NCBI BLAST. **Table S3.** Variation in structure and physical features of Galloanserae TLR. Information of amino protein length, molecular weight, charge, number of LRR and signal peptide length in Galloanserae TLR. **Table S4.** Identity in TLR secondary structures within Galloanserae. Whole protein, extracellular domain and intracellular domain identity of secondary structure motives within alignment of Galloanserae TLR. **Table S5.** List of binding residues identified in other vertebrates (fish and mammals) and their conservation within the Galloanserae lineage. Variability of residues at the predicted functional sites in Galloaserae birds with the prediction of changes in amino acid binding features compared to humans, mice and fish. **Table S6.** PAML codeml site model test for positive selection within Galloanserae TLR. Detailed information on parameters of the PAML model tests for positive selection within Galloanserae TLR. **Table S7.** Positively selected sites identified by PAML and FUBAR. Summary of positively selected sites identified by PAML and FUBAR with details on their location and p-values. **Table S8.** Positively selected sites identified by PRIME. Summary of positively selected sites identified by PRIME analysis with details on their location, p-values and changing properties. **Table S9.** Evolutionarily non-conservative sites identified by ConSurf. Summary of evolutionarily non-conservative sites identified by the ConSurf analysis with details on their location and conservation scores. **Table S10.** Co-location of sites under positive selection in TLR4, TLR5 and TLR7. Agreement of sites under positive selection in TLR4, TLR5 and TLR7 identified in this study with the results obtained by other evolutionarily studies aimed at detecting selection in TLR in vertebrates. Additional file 2: Figure S1. Phylogeny of *TLR* genes within Galloanserae. Maximum likelihood phylogeny of nucleotide sequences of *TLR4* (a), *TLR5* (b) and *TLR7* (c) in Galloanserae species calculated in PHYML. **Figure S2.** Phylogenetic relationships between the investigated species. Phylogenetic tree of the investigated species constructed based on consensus avian phylogenetic tool. **Figure S3.** Secondary structure alignment of Galloanserae TLR. Alignment of secondary structure motifs (α-helices, β-sheets and connecting sequences) in Galloanserae TLR. **Figure S4.** 3D structural similarity of Galloanserae TLR tertiary structures. Phenograms representing the 3D structural similarity of models of Galloanserae TLR tertiary structures. **Figure S5.** Species-specific differences in surface electrostatic potential. Visualisation of species-specific differences in surface electrostatic potential on 3D tertiary structure models. Additional file 3: Section S1. Variability in Galloanserae TLR basic protein features. Description of basic protein features in Galloanserae TLR. **Section S2.** Evolution of TLR secondary structures within the Galloanserae lineage. Description of secondary structure similarities in Galloanserae TLR. **Section S3.** Variability in features of predicted ligand-binding residues. Detailed information on amino acid binding features at the functional sites known in other vertebrates. **Section S4.** Analysis of positive selection. Detailed information and discussion on the results of the positive selection analysis.
