# Supplementary material for: Protein evolution of Toll-like receptors 4, 5 and 7 within Galloanserae birds
Source: Genet Sel Evol. 2014 Nov 12;46(1):72. doi: 10.1186/s12711-014-0072-6 (PMC4228102; doi:10.1186/s12711-014-0072-6)
Supplement: Additional file 1: Table S1. — Specification of sequences obtained from NCBI GenBank. Detailed information on NCBI GenBank sequences analysed in this study. Table S2. Homology of GaGaTLRs with other Galloanserae TLR molecules. Nucleotide and amino acid identity and similarity calculated in NCBI BLAST. Table S3. Variation in structure and physical features of Galloanserae TLR. Information of amino protein length, molecular weight, charge, number of LRR and signal peptide length in Galloanserae TLR. Table S4. Identity in TLR secondary structures within Galloanserae. Whole protein, extracellular domain and intracellular domain identity of secondary structure motives within alignment of Galloanserae TLR. Table S5. List of binding residues identified in other vertebrates (fish and mammals) and their conservation within the Galloanserae lineage. Variability of residues at the predicted functional sites in Galloaserae birds with the prediction of changes in amino acid binding features compared to humans, mice and fish. Table S6. PAML codeml site model test for positive selection within Galloanserae TLR. Detailed information on parameters of the PAML model tests for positive selection within Galloanserae TLR. Table S7. Positively selected sites identified by PAML and FUBAR. Summary of positively selected sites identified by PAML and FUBAR with details on their location and p-values. Table S8. Positively selected sites identified by PRIME. Summary of positively selected sites identified by PRIME analysis with details on their location, p-values and changing properties. Table S9. Evolutionarily non-conservative sites identified by ConSurf. Summary of evolutionarily non-conservative sites identified by the ConSurf analysis with details on their location and conservation scores. Table S10. Co-location of sites under positive selection in TLR4, TLR5 and TLR7. Agreement of sites under positive selection in TLR4, TLR5 and TLR7 identified in this study with the results obtained by other evolutionarily studies ai [file 12711_2014_72_MOESM1_ESM.pdf]

Title: **Protein evolution of Toll-like receptors 4, 5 and 7 within Galloanserae birds**

Authors: Michal Vinkler, Hana Bainová & Josef Bryja

Document: Additional file 1

Table S1 - Specification of sequences obtained from NCBI GenBank.

Gene	Species	Acronym	GenBank ID
TLR4	*Gallus gallus*	GaGaTLR4	AY064697.1
TLR4	*Gallus lafayetii*	GaLaTLR4	FJ915482.1
TLR4	*Gallus sonneratii*	GaSoTLR4	FJ915508.1
TLR4	*Gallus varius*	GaVaTLR4	FJ915504.1
TLR4	*Meleagris gallopavo*	MeGaTLR4	XM_003211211.1
TLR4	*Perdix perdix*	PePeTLR4	JQ713172.1
TLR4	*Anas platyrhynchos*	AnPlTLR4	JN048668.1
TLR4	*Anser anser*	AnAnTLR4	HQ436371.1
TLR5	*Gallus gallus*	GaGaTLR5	FJ915551.1
TLR5	*Gallus lafayetii*	GaLaTLR5	FJ915530.1
TLR5	*Meleagris gallopavo*	MeGaTLR5	HQ436463.1
TLR5	*Phasianus colchicus*	PhCoTLR5	JF767220.1
TLR5	*Numida meleagris*	NuMeTLR5	JF767221.1
TLR5	*Perdix perdix*	PePeTLR5	JQ713180.1
TLR5	*Anas platyrhynchos*	AnPlTLR5	KF255555.1
TLR5	*Tadorna tadorna*	TaTaTLR5	KF255554.1
TLR5	*Anser anser*	AnAnTLR5	JN641303.1
TLR7	*Gallus gallus*	GaGaTLR7	AJ632302.1
TLR7	*Gallus lafayetii*	GaLaTLR7	FJ915556.1
TLR7	*Gallus sonneratii*	GaSoTLR7	FJ915580.1
TLR7	*Gallus varius*	GaVaTLR7	FJ915576.1
TLR7	*Meleagris gallopavo*	MeGaTLR7	XM_003203086.1
TLR7	*Coturnix japonica*	CoJaTLR7	AB553582.1
TLR7	*Perdix perdix*	PePeTLR7	JQ713178
TLR7	*Anas platyrhynchos*	AnPlTLR7	DQ888644.1
TLR7	*Anser cygnoides*	AnCyTLR7	JQ910168.1

In TLR7 only the short splicing variants according to the sequence GenBank:AJ632302.1 were used, see Philbin et al. (2005). In phylogenetic and structural analysis we used the following human and mouse sequences as outgroups: *Homo sapiens Tlr4* (*HoSaTlr4*) GenBank:NM_138554.4, *HoSaTlr5* GenBank:NM_003268.5, *HoSaTlr7* GenBank:NM_016562.3, *Mus musculus* (*MuMuTlr4*) GenBank:NM_021297.2, *MuMuTlr5* GenBank:NM_016928.2, *MuMuTlr7* GenBank:NM_133211.3 and their protein equivalents.

Table S2 - Homology of GaGaTLRs with other Glloanserae TLR molecules.

Nucleotide (Nt) and amino acid (Aa) identity and similarity (Aa positives; all values given in %) were calculated in NCBI BLAST.

	Nt identity	Aa identity	Aa positives
**GaGaTLR4**			
GaSoTLR4	99	99	100
GaLaTLR4	99	99	100
GaVaTLR4	99	99	99
PePeTLR4	96	95	97
MeGaTLR4	95	93	97
AnAnTLR4	87	82	90
AnPlTLR4	87	82	89
**GaGaTLR5**			
GaLaTLR5	100	100	100
MeGaTLR5	98	96	97
PhCoTLR5	96	95	97
PePeTLR5	95	93	96
NuMeTLR5	94	93	95
AnAnTLR5	88	83	90
AnPlTLR5	87	81	88
TaTaTLR5	87	81	88
**GaGaTLR7**			
GaSoTLR7	99	99	99
GaLaTLR7	99	99	99
GaVaTLR7	99	99	99
MeGaTLR7	95	94	96
PePeTLR7	95	93	96
CoJaTLR7	95	94	97
AnCyTLR7	89	86	92
AnPlTLR7	88	86	91

Table S3 - Variation in structure and physical features of Galloanserae TLRs.

TLR	Aa length	Mol. Weight (Da)	Charge at pH7	No. of LRRs	Signal peptide
GaGaTLR4	843	110.85	1.3	22	1-30
GaLaTLR4	843	110.79	2.3	21	1-30
GaSoTLR4	843	110.81	2.5	21	1-30
GaVaTLR4	843	110.826	2.5	21	1-30
PePeTLR4	843	110.887	3.7	18	1-30
MeGaTLR4	843	110.741	4.1	19	1-30
AnAnTLR4	843	111.253	6.7	18	1-30
AnPlTLR4	843	111.182	2.5	16	1-30
GaGaTLR5	861	114.717	-2.0	22	1-21
GaLaTLR5	861	114.717	-2.0	22	1-21
MeGaTLR5	862	114.689	-2.4	19	1-16
PhCoTLR5	861	114.594	-2.0	19	1-21
PePeTLR5	862	114.241	-3.2	19	1-20
NuMeTLR5	861	114.156	-4.0	19	1-21
AnAnTLR5	860	114.348	-3.8	17	1-21
AnPlTLR5	859	114.276	-0.8	16	1-16
TaTaTLR5	859	114.594	-2.0	18	1-21
GaGaTLR7	1047	139.8	18.6	27	1-25
GaSoTLR7	1047	139.77	18.6	27	1-25
GaLaTLR7	1047	139.77	18.6	27	1-25
GaVaTLR7	1047	139.77	18.6	27	1-25
MeGaTLR7	1047	139.153	23.6	27	1-25
PePeTLR7	1047	138.974	20.6	27	1-25
CoJaTLR7	1047	139.158	17.2	26	1-25
AnCyTLR7	1047	139.433	22.4	27	1-25
AnPlTLR7	1047	139.354	16.6	27	1-25

Table S4 - Identity in TLR secondary structures within Galloanserae.

GaGaTLR sequences were taken as reference sequences. In TLR7 the analysis was performed with sequences after endoplasmic reticulum cleavage. ECD = extracellular domain, ICD = intracellular domain.

TLR	Whole protein identity	ECD identity	ICD identity
**GaGaTLR4**			
GaSoTLR4	817/843 (96.9%)	616/639 (96.4%)	178/181 (98.3%)
GaLaTLR4	813/843 (96.4%)	616/639 (96.4%)	174/181 (96.1%)
GaVaTLR4	821/843 (97.4%)	623/639 (97.5%)	175/181 (96.7%)
PePeTLR4	804/843 (95.4%)	613/639 (95.9%)	168/181 (92.8%)
MeGaTLR4	801/843 (95.0%)	608/639 (95.1%)	170/181 (93.9%)
AnAnTLR4	775/843 (91.9%)	579/639 (90.6%)	173/181 (95.6%)
AnPlTLR4	776/843 (92.1%)	580/639 (90.8%)	174/181 (96.1%)
**GaGaTLR5**			
GaLaTLR5	859/859 (100.0%)	641/641 (100.0%)	200/200 (100.0%)
PePeTLR5	831/859 (96.7%)	615/641 (95.9%)	198/200 (99.0%)
MeGaTLR5	818/859 (95.2%)	608/641 (94.9%)	192/200 (96.0%)
PhCoTLR5	816/859 (95.0%)	604/641 (94.2%)	194/200 (97.0%)
NuMeTLR5	822/859 (95.7%)	607/641 (94.7%)	197/200 (98.5%)
AnAnTLR5	824/859 (95.9%)	610/641 (95.2%)	196/200 (98.0%)
**GaGaTLR7**			
GaSoTLR7	604/619 (97.6%)	403/414 (97.3%)	186/190 (97.9%)
GaLaTLR7	604/619 (97.6%)	403/414 (97.3%)	186/190 (97.9%)
GaVaTLR7	604/619 (97.6%)	403/414 (97.3%)	186/190 (97.9%)
PePeTLR7	578/619 (93.4%)	395/414 (95.4%)	170/190 (89.5%)
MeGaTLR7	573/619 (92.6%)	379/414 (91.5%)	181/190 (95.3%)
CoJaTLR7	578/619 (93.4%)	384/414 (92.8%)	181/190 (95.3%)
AnPlTLR7	574/619 (92.7%)	387/414 (93.5%)	174/190 (91.6%)

Table S5 - List of binding residues identified in other vertebrates (fish and mammals) and their conservatism within Galloanserae lineage. The GaGaTLR position numbering has been adopted. References are cited as follows: 1 - Kim et al. (2007), 2 - Park et al. (2009), 3 - Ohto et al. (2012), 4 - Walsh et al. (2008), 5 - Yoon et al. (2012), 6 - Andersen-Nissen et al. (2007), 7 - Wei et al. (2009).

TLR	Site	Residue function	Ref.	Galloaserae residue conservatism	HoSa	MuMu	DaRe	Prediction of changes in amino acid binding features
TLR4	33	MD-2 dimerization	1	uniformly C	C29	C28	—	preserved
TLR4	44	MD-2 dimerization	1	uniformly C	C40	C39	—	preserved
TLR4	46	MD-2 dimerization	1	uniformly G	E42	D41	—	probably altered
TLR4	88	MD-2 dimerization	1	uniformly D	D84	D83	—	preserved
TLR4	139	MD-2 dimerization	1	uniformly E	E135	E134	—	preserved
TLR4	163	MD-2 dimerization	1	uniformly H	H159	H158	—	preserved
TLR4	238	MD-2 dimerization	1	uniformly R	R234	R233	—	preserved
TLR4	268	LPS and MD-2 binding	1	Galliformes mostly T, MeGa S, Aseriformes R	R264	K263	—	probably altered in Galliformes
TLR4	293	MD-2 dimerization	1	uniformly V	R289	R288	—	probably altered
TLR4	345	LPS binding	2	mostly K (MeGa R)	K341	Q339	—	preserved
TLR4	369	LPS binding	2	uniformly K	K362	K360	—	preserved
TLR4	372	TLR dimerization	2	uniformly N	N365	I363	—	preserved
TLR4	376	lipid IVa recognition	3	uniformly Q	E369	K367	—	probably altered
TLR4	393	LPS binding	2,4	uniformly R	G384	A382	—	as in horse
TLR4	395	TLR dimerization	2	uniformly S	S386	S384	—	preserved
TLR4	397	LPS binding	2	Galliformes uniformly L, Aseriformes T	K388	S386	—	altered
TLR4	420	TLR dimerization	2	uniformly D	V411	A409	—	probably altered
TLR4	424	LPS binding	3	uniformly T	S415	S413	—	probably preserved
TLR4	425	MD-2 dimerization	2	uniformly G	S416	A414	—	possibly preserved
TLR4	426	MD-2 dimerization	2	mostly D (order *Gallus* E)	N417	N415	—	possibly preserved
TLR4	428	MD-2 dimerization	3	Galliformes uniformly A, Aseriformes T	L419	M417	—	probably altered
TLR4	442	TLR dimerization	2	uniformly K	N433	T431	—	possibly preserved

TLR4	445	LPS binding	2	Galliformes uniformly H, AnAn D, AnPl N	Q436	R434	—	possibly preserved
TLR4	448	MD-2 dimerization	2	uniformly T	E439	E437	—	possibly preserved
TLR4	449	LPS and MD-2 binding	2	uniformly Y	F440	F438	—	altered
TLR4	453	LPS and MD-2 binding	2,3	uniformly L	L444	L442	—	preserved
TLR4	454	MD-2 dimerization	3	Galliformes uniformly L, Anseriformes S	S445	S443	—	probably altered
TLR4	472	LPS and MD-2 binding	2	uniformly S	F463	F461	—	altered
TLR4	516	TLR dimerization	2	uniformly K	Q507	Q505	—	possibly preserved
TLR5	33	FLA binding	5	Galliformes uniformly M, AnAn V, AnPl&TaTa M	F32	F32	I33	probably preserved
TLR5	35	FLA binding	5	Galliformes mostly N (NuMe S), AnAn Y, AnPl&TaTa N	R34	R34	I35	probably altered
TLR5	36	FLA binding	5	mostly S (PhCo F)	F35	G35	I35	probably altered
TLR5	37	FLA binding	5	uniformly C	C36	C36	R37	probably altered
TLR5	53	FLA binding	5	Galliformes uniformly F, Anseriformes uniformly L	L52	L53	D53	probably altered
TLR5	55	FLA binding	5	Galliformes uniformly T, AnAn S, AnPl&TaTa N	S54	S55	S55	preserved in Galliformes and AnAn
TLR5	56	FLA binding	5	Galliformes uniformly Y, Anseriformes uniformly F	F55	F56	L56	probably altered
TLR5	77	FLA binding	5	uniformly E	E76	E77	K77	probably altered
TLR5	79	FLA binding	5	uniformly G	G78	G79	E79	probably altered
TLR5	80	FLA binding	5	mostly T (AnPl&TaTa S)	S79	T80	Q80	probably altered
TLR5	106	FLA binding	5	Galliformes uniformly F, AnAn Y, AnPl&TaTa Q	S104	Q105	Y105	probably preserved
TLR5	130	FLA binding	5	mostly Q (NuMe R, AnPl&TaTa H)	F128	S129	Q129	preserved (except for NuMe&AnPl&TaTa)
TLR5	156	FLA binding	5	uniformly G	K154	G155	D155	probably altered
TLR5	181	FLA binding	5	uniformly F	S179	F180	F180	preserved
TLR5	183	FLA binding	5	Galliformes uniformly K, AnAn A, AnPl&TaTa D	Q181	Q182	K182	bond preserved in Galliformes
TLR5	209	FLA binding	5	GaGa,GaLa,MeGa&NuMe T, PePe,PhCo&AnAn S, AnPl&TaTa Y	S207	K208	T208	preserved (except for AnPl&TaTa)

TLR5	211	FLA binding	5	uniformly Y	Y209	F210	Q210	probably altered
TLR5	214	FLA binding	5	mostly D (AnPl&TaTa N)	V212	V213	N213	possibly preserved (AnPl&TaTa preserved)
TLR5	gap	FLA binding	5	position missing in Amniotes	—	—	Y215	—
TLR5	241	FLA binding	5	mostly S (NuMe N)	T239	T240	K242	probably altered
TLR5	gap	FLA binding	5	position missing in Amniotes	—	—	N265	—
TLR5	265	FLA binding	5	uniformly H	H263	H264	Y267	probably preserved
TLR5	266	FLA binding	5	mostly T (PhCo I)	I264	I265	N268	probably altered
TLR5	268	FLA binding	5	uniformly G	G266	G267	G270	preserved
TLR5	269	FLA binding	5,6	uniformly S	A267	P268	S271	preserved
TLR5	270	FLA binding	5	uniformly G	G268	G269	S272	possibly preserved
TLR5	271	FLA binding, TLR dimerization	5	uniformly F	F269	F270	F273	preserved
TLR5	272	Pred. FLA binding	6	uniformly G	G270	G271	G274	preserved
TLR5	273	FLA binding	5	mostly F (AnPl&TaTa Y)	F271	F272	H275	possibly preserved
TLR5	274	FLA binding	5	uniformly N	H272	Q273	T276	probably altered
TLR5	275	FLA binding	5	uniformly N	N273	N274	N277	preserved
TLR5	276	FLA binding	5	uniformly L	I274	I275	F278	possibly preserved
TLR5	277	FLA binding	5	uniformly K	K275	R276	K279	preserved
TLR5	296	Pred. FLA binding	6	uniformly D	D294	D295	D298	preserved
TLR5	298	Pred. FLA binding	6	uniformly S	S296	S297	S300	preserved
TLR5	301	FLA binding	5	Galliformes mostly F (NuMe Y), Anseriformes uniformly Y	F299	F300	K303	probably altered
TLR5	320	Pred. FLA binding	6	uniformly N	N318	N319	T322	preserved
TLR5	322	Pred. FLA binding	6	mostly F (AnAn S)	A320	A321	A324	probably altered
TLR5	344	Pred. FLA binding	6	uniformly N	N342	N343	N346	preserved
TLR5	346	Pred. FLA binding	6	uniformly S	S344	S345	S348	preserved
TLR5	347	TLR dimerization	5	uniformly S	Y345	Y346	Q349	probably altered
TLR5	348	TLR dimerization	5	uniformly N	N346	N347	N350	preserved
TLR5	349	TLR dimerization	5	uniformly L	L347	L348	F351	possibly preserved
TLR5	352	FLA binding	5	uniformly E	E350	E351	S354	probably altered

TLR5	354	FLA binding	5	uniformly Y	Y352	Y353	D356	probably altered
TLR5	367	Pred. FLA binding	6	uniformly I	I365	V366	I369	preserved
TLR5	368	Pred. FLA binding	6	uniformly D	D366	D367	D370	preserved
TLR5	371	TLR dimerization	5	uniformly Q	K369	R370	Y373	probably altered
TLR5	373	TLR dimerization	5	uniformly H	H371	H372	H375	preserved
TLR5	375	TLR dimerization	5	uniformly G	A373	G374	R377	probably altered
TLR5	376	FLA binding	5	uniformly M	I374	I375	A378	possibly preserved
TLR5	378	FLA binding	5	mostly G (TaTa D)	Q376	Q377	G380	preserved (except for TaTa)
TLR5	379	FLA binding	5	GaGa,GaLa,MeGa&NuMe E,PePe,PhCo Q, Anseriformes uniformly Q	D377	D378	D381	preserved (probably altered in PePe, PhCo and Anseriformes)
TLR5	380	FLA binding	5	uniformly K	Q378	Q379	Q382	probably altered
TLR5	391	Pred. FLA binding	6	uniformly I	L389	L390	L393	preserved
TLR5	392	Pred. FLA binding	6	uniformly D, AnAn N	D390	D391	N394	probably altered in AnAn
TLR7	500	Pred. ssRNA binding	7	uniformly R	K502	R503	—	preserved
TLR7	502	Pred. ssRNA binding	7	uniformly N	S504	N505	—	possibly altered
TLR7	524	Pred. ssRNA binding	7	uniformly G	G526	G527	—	preserved
TLR7	529	Pred. ssRNA binding	7	uniformly Q	Q531	Q532	—	preserved
TLR7	549	Pred. ssRNA binding	7	uniformly N	N551	N552	—	preserved
TLR7	551	Pred. ssRNA binding	7	uniformly R	R553	R554	—	preserved
TLR7	554	Pred. ssRNA binding	7	uniformly L	L556	L557	—	preserved
TLR7	573	Pred. ssRNA binding	7	uniformly N	S575	S576	—	possibly altered
TLR7	576	Pred. ssRNA binding	7	uniformly H	H578	H579	—	preserved

Table S6 - PAML codeml site model test for positive selection within Galloanserae TLRs.

Models: H0: M8a (neutral) or H1: M8 (alternative); degrees of freedom (df) = np1-np0; lnL = log-likelihood; LRT = Likelihood ratio test (2×(lnH1 - lnH0)); κ (ts/tv) = gene kappa estimate; transition/transversion rate; ω (dN/dS >1) = gene omega estimate, non-synonymous/synonymous substitution rate > 1, which is indicative of positive selection; p1 = proportion of sites dN/dS>1, i.e. the proportion of sites having ω > 1.

TLR	Model	Parameters	df	lnL	LRT	p-value	κ (ts/tv)	ω (dN/dS >1)	p1
TLR4	M8a	18	1	-5974.214632	0.405759233	0.476	3.53774	1.48815	0.08782
	M8	19		-5973.869036					
TLR5	M8a	20	1	-7013.825542	0.000005616	< 0.001	3.86786	3.59096	0.05294
	M8	21		-7003.518166					
TLR7	M8a	20	1	-7437.332061	0.001967845	0.035	3.57988	4.18087	0.01876
	M8	21		-7432.542416					

Table S7 - Positively selected sites identified by PAML and FUBAR.

The GaGaTLR position numbering has been adopted. ECD = extracellular domain, TMD = transmembrane domain, ICD = intracellular domain.

TLR	Site	Domain	Structural region	Functional position	PAML (p)	FUBAR (p)
TLR4			No evidence for positive or diversifying selection			
TLR5	209	ECD	LRR7 region	Flagellin-binding site identified in fish	—	0.052
TLR5	281	ECD	LRR9 region	—	0.001	0.048
TLR5	341	ECD	LRR12 region	—	0.008	0.059
TLR5	342	ECD	LRR12 region	Flagellin-binding region (prox. to res. 344)	—	0.048
TLR5	468	ECD	LRR17 region	—	0.072	0.049
TLR5	510	ECD	LRR19 region	—	0.089	—
TLR5	647	TMD	TMD	Membrane region	0.074	—
TLR7	26	ECD	Signal peptide	Expression guidance site	0.024	—
TLR7	99	ECD	LRR2 region	Excised region	0.048	0.035
TLR7	144	ECD	LRR3 region	Excised region	0.069	0.051
TLR7	155	ECD	LRR4 region	Excised region	0.056	—
TLR7	537	ECD	LRR16 region	—	0.055	0.032
TLR7	638	ECD	LRR20 region	—	0.033	—
TLR7	748	ECD	LRR25 region	—	0.045	—

Table S8 - Positively selected sites identified by PRIME.

Amino acid properties categorised according to Atchley et al. (2005). The GaGaTLR position numbering has been adopted. ECD = extracellular domain.

TLR	Site	Domain	Structural region	Functional position	PRIME	Changing properties
TLR4	343	ECD	Connective region	LPS-binding region (prox. to res. 345 and 369)	p=0.080	Refractivity/Heat Capacity
TLR5	180	ECD	LRR5 region	Flagellin-binding region identified in fish (prox. to res. 181)	p=0.095	Refractivity/Heat Capacity
TLR5	259	ECD	LRR6 region	—	p=0.004	Refractivity/Heat Capacity
TLR5	379	ECD	LRR13 region	Flagellin-binding site identified in fish	p=0.061	Volume
TLR5	422	ECD	LRR15 region	—	p=0.092	Charge/ Iso-electric point
TLR5	510	ECD	LRR19 region	—	p=0.025	Refractivity/Heat Capacity
TLR7	99	ECD	LRR2 region	Excised region	p=0.092	Secondary structure factor
TLR7	669	ECD	LRR21 region	—	p=0.024	Refractivity/Heat Capacity

Table S9 - Evolutionarily non-conservative sites identified by ConSurf.

Only sites with grade 1 (i.e. the least conservative sites detected based on the conservation score) are shown. Unreliable positions (i.e. those in which the estimated score interval spans 4 or more grades) were excluded. The GaGaTLR position numbering has been adopted.

TLR	Site	Variable residues	Conservation score	Confidence interval	Structural region	Functional position
TLR4	8	T,I,L	1.824	0.156, 2.712	Signal peptide	Expression guidance site
TLR4	10	Q,W,L	2.978	0.156, 2.712	Signal peptide	Expression guidance site
TLR4	13	R,G,V	12.21	0.926,15.420	Signal peptide	Expression guidance site
TLR4	14	G,E,V	3.671	0.156, 6.712	Signal peptide	Expression guidance site
TLR4	17	Q,R	2.13	0.156, 2.712	Signal peptide	Expression guidance site
TLR4	19	A,L,V	2.288	0.156, 2.712	Signal peptide	Expression guidance site
TLR4	40	T,R,K	2.424	0.173, 2.827	Connective region	N-terminal MD-2-binding region
TLR4	205	A,T,G	1.596	0.173, 2.827	LRR6 region	—
TLR4	246	A,D,V	2.93	0.173, 2.827	LRR8 region	—
TLR4	268	S,T,R	1.85	0.173, 2.827	LRR9 region	MD-2 dimerization and LPS-binding site
TLR4	270	S,N,I	2.28	0.173, 2.827	LRR9 region	MD-2-binding region (prox. to res. 268)
TLR4	273	M,I,L,V	2.882	0.173, 2.827	LRR9 region	—
TLR4	274	T,K,G,E	7.104	0.973, 6.980	LRR9 region	—
TLR4	275	A,D,E	1.887	0.173, 2.827	LRR9 region	—
TLR4	301	D,E	3.6	0.173, 2.827	LRR10 region	—
TLR4	383	H,S,Y	12.68	0.973, 6.980	LRR14 region	—
TLR4	406	K,R,G	2.955	0.173, 2.827	LRR14 region	—
TLR4	444	D,I	1.705	0.173, 2.827	LRR16 region	TLR-dimerization and LPS-binding region (prox. to res. 442 and 445)
TLR4	471	M,I,K	2.405	0.173, 2.827	LRR17 region	MD-2 dimerization and LPS-binding region (prox. to res. 472)
TLR4	512	I,L,V	1.967	0.173, 2.827	LRR19 region	TLR-dimerization region (prox. to res. 516)
TLR4	519	Q,W,E	4.072	0.173, 6.980	LRR19 region	—

TLR4	521	D,Y,V	13.2	0.973,16.030	LRR19 region	—
TLR4	627	M,L,V	1.862	0.173, 2.827	LRRCT region	—
TLR4	655	G,L,V	4.667	0.926, 6.712	TMD	Membrane region
TLR4	671	F,S,I	2.291	0.156, 2.712	Connective region	—
TLR4	686	T,D,P	3.084	0.156, 2.712	Connective region	—
TLR5	12	R,G,E	2.008	0.165, 2.517	Signal peptide	Expression guidance site
TLR5	82	H,R,Y	2.034	0.094, 2.128	LRR2 region	Flagellin-binding region identified in fish (prox. to res. 80)
TLR5	87	N,I,Y	1.445	0.094, 2.128	LRR2 region	—
TLR5	125	A,I,E,V	1.847	0.094, 2.128	LRR4 region	—
TLR5	183	A,D,K	1.221	0.094, 2.128	LRR6 region	Flagellin-binding site identified in fish
TLR5	207	S,T,D,Y	1.523	0.094, 2.128	LRR7 region	Flagellin-binding region identified in fish (prox. to res. 209)
TLR5	209	S,T,Y	1.68	0.094, 2.128	LRR7 region	Flagellin-binding site identified in fish
TLR5	216	T,M,V	0.927	0.094, 0.707	LRR7 region	Flagellin-binding region identified in fish (prox. to res. 214)
TLR5	259	T,N,Y	1.247	0.094, 2.128	LRR9 region	—
TLR5	264	S,F,L	2.53	0.094, 2.128	LRR9 region	Flagellin-binding region (prox. to res. 265, 269, 296 and 298)
TLR5	281	Q,T,D,N,K,E	11	0.707, 5.309	LRR9 region	—
TLR5	293	H,Q,R	1.587	0.094, 2.128	LRR10 region	—
TLR5	314	R,G	1.606	0.094, 2.128	LRR10 region	—
TLR5	341	M,T,R,K,E	18.34	0.707, 5.309	LRR12 region	—
TLR5	342	F,T,I,V	1.318	0.094, 2.128	LRR12 region	Flagellin-binding region (prox. to res. 344)
TLR5	413	S,G	1.247	0.094, 2.128	LRR15 region	—
TLR5	422	H,D,G	1.521	0.094, 2.128	LRR15 region	—
TLR5	451	D,G	1.919	0.094, 2.128	LRR17 region	—
TLR5	456	F,S,L	4.889	0.094, 2.128	LRR17 region	—
TLR5	468	S,H,T,R	1.574	0.094, 2.128	LRR17 region	—
TLR5	508	F,I,V	1.288	0.094, 2.128	LRR19 region	—
TLR5	510	H,F,L	1.886	0.094, 2.128	LRR19 region	—
TLR5	525	S,N,R	1.471	0.094, 2.128	LRR19 region	—
TLR5	532	R,I,G	1.749	0.094, 2.128	LRR20 region	—
TLR5	546	R,G	1.606	0.094, 2.128	LRR20 region	—

TLR5	625	F,L	1.238	0.094, 2.128	LRRCT region		—
TLR5	648	T,I,L	2.366	0.165, 2.517	TMD	Membrane region	
TLR5	659	A,T,V	2.583	0.165, 2.517	TMD	Membrane region	
TLR7	3	H,Q,R,P	5.542	1.538,10.100	Signal peptide	Expression guidance site	
TLR7	26	H,A,N,R,Y	8.085	1.538,10.100	LRRNT region	Excised region	
TLR7	39	S,F	3.311	0.398, 4.181	LRRNT region	Excised region	
TLR7	82	A,T,I	2.589	0.398, 4.181	LRR1 region	Excised region	
TLR7	99	A,P,V	10.61	1.538,10.100	LRR2 region	Excised region	
TLR7	144	S,A,T,D,P	10.33	1.538,10.100	LRR3 region	Excised region	
TLR7	155	S,N,K,R	3.998	0.398, 4.181	LRR4 region	Excised region	
TLR7	272	N,I,V	2.324	0.398, 4.181	LRR8 region	Excised region	
TLR7	298	Q,R	2.484	0.398, 4.181	LRR9 region	Excised region	
TLR7	313	D,N,E	2.262	0.398, 4.181	LRR10 region	Excised region	
TLR7	383	R,G,E	12.44	1.538,10.100	LRR12 region	Excised region	
TLR7	386	F,D,Y	3.706	0.398, 4.181	LRR12 region	Excised region	
TLR7	390	S,N,K	2.98	0.398, 4.181	LRR12 region	Excised region	
TLR7	437	F,S,I	3.78	0.452, 4.785	LRR14 region		—
TLR7	509	S,I,V	3.458	0.452, 4.785	LRR15 region		—
TLR7	537	S,H,Y	5.233	0.452, 4.785	LRR16 region		—
TLR7	565	F,Y,L	2.828	0.452, 4.785	LRR18 region		—
TLR7	638	A,T,N,K	6.603	0.452, 4.785	LRR20 region		—
TLR7	665	S,P,Y	6.603	0.452,11.560	LRR21 region		—
TLR7	700	T,K,I,V	5.108	0.452, 4.785	LRR23 region		—
TLR7	732	H,R	2.935	0.452, 4.785	LRR24 region		—
TLR7	746	Q,K,G	4.14	0.452, 4.785	LRR25 region		—
TLR7	748	S,Q,T,K	3.699	0.452, 4.785	LRR25 region		—
TLR7	794	W,R,G	6.487	0.452,11.560	LRRCT region		—
TLR7	919	S,A,T,P	4.097	0.398, 4.181	TIR domain	Signalling region	

Table S10 – Co-location of sites under positive selection in TLR4, TLR5 and TLR7 identified in this study with the results obtained by other evolutionarily studies aimed at detection of selection in TLRs in vertebrates.

References: 1 - Vinkler et al. (2009); 2 - Wlasiuk et al. (2009); 3 - Wlasiuk and Nachman (2010); 4 - Alcaide and Edwards (2011); 5 - Areal et al. (2011); 6 - Smith et al. (2012); 7 - Fornuskova et al. (2013); 8 - Grueber et al. (2014). The GaGaTLR position numbering has been adopted.

TLR	Selection	ConSurf	Ref.1	Ref.2	Ref.3	Ref.4	Ref.5	Ref.6	Ref.7	Ref.8	Consensus	Function
TLR4		8					8 (4)					
TLR4		10					9 (5)					
TLR4		13					13 (9)					
TLR4		14										
TLR4		17										
TLR4		19										
TLR4		40										
TLR4							60 (56)					
TLR4							62 (58)					
TLR4					79 (75)							
TLR4					100 (96)							
TLR4							124 (120)					
TLR4					143 (139)							
TLR4							165 (161)					
TLR4					188 (184)							
TLR4					190 (186)							
TLR4							193 (189)					
TLR4							197 (193)					
TLR4		205			205 (201)						205	—
TLR4							207 (203)					
TLR4					208 (204)		208 (204)				[208]	
TLR4			220		220 (216)					221	[220]	
TLR4					233 (229)							

TLR4	246	244			244 (240)		246	246	—
TLR4							250		
TLR4					254 (250)				
TLR4	268								
TLR4	270						270	270	MD-2-binding region (prox. to res. 268)
TLR4		271 (270)					271	[271]	
TLR4		272 (271)							
TLR4	273	273 (272)	273 (269)					273	—
TLR4	274	274 (273)		274	274 (270)		274	274	—
TLR4	275	275 (274)	275 (271)		275 (271)			275	—
TLR4		278 (277)	278 (274)			278 (273)		[278]	
TLR4		280 (279)			280 (276)			[280]	
TLR4		294 (293)	296 (292)		294 (290)			[294]	
TLR4		297 (296)							
TLR4		299 (298)	299 (295)		299 (295)			[299]	
TLR4			300 (296)						
TLR4	301	301 (300)	301 (297)				301	301	—
TLR4			302 (298)		302 (298)		302	[302]	
TLR4			303 (299)						
TLR4		304 (303)	304 (300)		304 (300)			[304]	
TLR4		306 (305)			305 (301)				
TLR4		307 (306)							
TLR4			312 (308)						
TLR4		316 (315)							
TLR4		321 (320)			321 (317)			[321]	
TLR4		322 (321)							
TLR4		323 (322)	323 (319)		323 (319)		323	[323]	
TLR4		325 (324)	325 (321)		325 (321)			[325]	
TLR4		326 (325)	326 (322)		326 (322)			[326]	
TLR4			327 (323)		327 (323)			[327]	

TLR4			329 (328)	328 (324)		328 (324)			[328]	
TLR4						329 (325)				
TLR4			333 (332)	331 (327)		333 (329)		333	[333]	
TLR4				335 (331)						
TLR4			338 (337)							
TLR4			341 (340)			340 (336)	341 (335)		[341]	
TLR4						342 (338)				
TLR4	343		345 (344)			346 (342)		345	343	LPS-binding region (prox. to res. 345 and 369)
TLR4			347 (346)							
TLR4			348 (347)							
TLR4							351 (345)			
TLR4					352	353 (349)		352	[352]	
TLR4			354 (353)	353 (349)			353 (347)		[353]	
TLR4			356 (355)	355 (351)		355 (351)			[355]	
TLR4			358 (357)	358 (354)					[358]	
TLR4			359 (358)			360 (356)				
TLR4			364 (363)			364 (357)		365	[364]	
TLR4			367 (366)	367 (360)		367 (360)			[367]	
TLR4			370 (369)			370 (363)	370 (361)	370	[370]	
TLR4			372 (371)	372 (365)		371 (364)	372 (363)		[372]	
TLR4			373 (372)							
TLR4			375 (374)	375 (368)		375 (368)	375 (366)	375	[375]	
TLR4			377 (376)			377 (370)	377 (368)		[377]	
TLR4			378 (377)	378 (371)		378 (371)			[378]	
TLR4			379 (378)							
TLR4		383								
TLR4								387		
TLR4			393 (392)			391 (382)				
TLR4			397 (396)		397			397	[397]	
TLR4			398 (397)					398	[398]	

TLR4		402 (401)					402	[402]	
TLR4		403 (402)	403 (394)		403 (394)			[403]	
TLR4		404 (403)							
TLR4		405 (404)			405 (396)	405 (394)		[405]	
TLR4	406			406			406	406	—
TLR4		409 (408)	411 (402)		409 (400)	409 (398)		[409]	
TLR4			419 (410)						
TLR4		422 (421)	424 (415)						
TLR4			432 (423)						
TLR4							435		
TLR4	444	445 (444)						444	LPS binding region
TLR4		446 (445)	446 (437)		446 (437)		447	[446]	
TLR4							448		
TLR4						453 (442)			
TLR4					456 (447)				
TLR4			459 (450)						
TLR4	471	469 (468)	469 (460)		469 (460)		472	471	MD-2 dimerization and LPS-binding region (prox. to res. 472)
TLR4		473 (472)							
TLR4		477 (476)	477 (468)		477 (468)			[477]	
TLR4		480 (479)	480 (471)		480 (471)	480 (469)		[480]	
TLR4			483 (474)						
TLR4			484 (475)		486 (474)				
TLR4		496 (495)	496 (487)		496 (487)			[496]	
TLR4			503 (494)		502 (493)				
TLR4			505 (496)						
TLR4		509 (508)			509 (500)			[509]	
TLR4	512	514 (513)	514 (505)		514 (505)			512	TLR-dimerization region (prox. to res. 516)
TLR4	519								
TLR4	521	523 (522)	523 (514)					521	—

TLR4		526 (525)	526 (517)			[526]	
TLR4			529 (520)	529 (520)		[529]	
TLR4			530 (521)				
TLR4		543 (542)	542 (533)				
TLR4			546 (537)	546 (537)		[546]	
TLR4			551 (542)	551 (542)		[551]	
TLR4			553 (544)				
TLR4			570 (561)				
TLR4			575 (566)				
TLR4		582 (581)					
TLR4		587 (586)					
TLR4		613 (612)		613 (604)		[613]	
TLR4		617 (616)	615 (606)				
TLR4			620 (611)				
TLR4		623 (622)		622 (613)			
TLR4	627		625 (616)	625 (616)		627	—
TLR4		633 (632)		631 (622)			
TLR4			636 (626)				
TLR4		639 (638)					
TLR4		640 (639)					
TLR4		643 (642)					
TLR4		649 (648)	649 (639)	649 (639)		[649]	
TLR4	655						
TLR4		657 (656)					
TLR4		658 (657)		658 (648)		[658]	
TLR4	671						
TLR4	686		687 (673)	687 (673)		686	—
TLR4				836 (822)	834 (818)		
TLR4			missing (833)				

TLR5		12							
TLR5			15 (14)		15 (14)			[15]	
TLR5			30 (29)						
TLR5						35 (34)			
TLR5					72 (71)				
TLR5		82							
TLR5		87							
TLR5			106 (104)			106 (104)		[106]	
TLR5		125							
TLR5					130 (128)				
TLR5					156 (154)				
TLR5			160 (158)						
TLR5			170 (168)		172 (170)				
TLR5	180								
TLR5		183	183 (181)					183	Flagellin-binding site identified in fish
TLR5			199 (197)						
TLR5		207							
TLR5	209	209	209 (207)		209 (207)		209	209	Flagellin-binding site identified in fish
TLR5							212		
TLR5							214		
TLR5		216					215	216	Flagellin-binding region identified in fish (prox. to res. 214)
TLR5			232 (230)						
TLR5							237		
TLR5				244			244	[244]	
TLR5	259	259		258			258	259	—
TLR5				261			261	[261]	
TLR5		264	264 (262)	264			264	264	Flagellin-binding region (prox. to res. 265, 269, 296 and 298)
TLR5							265		

TLR5			270 (268)				270 (268)		[270]	
TLR5								276		
TLR5								278		
TLR5	281	281						281	281	—
TLR5			282 (280)					282	[282]	
TLR5		293	**294 (292)**					293	293	—
TLR5							297 (295)	299		
TLR5						307 (305)		306		
TLR5					309		309 (307)	309	[309]	
TLR5		314	**314 (312)**						314	—
TLR5								322		
TLR5							328 (326)			
TLR5					331			331	[331]	
TLR5								332		
TLR5								335		
TLR5	341	341								
TLR5	342	342				342 (340)			342	Flagellin-binding region (prox. to res. 344)
TLR5			356 (354)							
TLR5								359		
TLR5			365 (363)							
TLR5	379							378	379	Flagellin-binding site identified in fish
TLR5						384 (382)		384	[384]	
TLR5							395 (393)			
TLR5								398		
TLR5			402 (400)	402 (400)		402 (400)			[402]	
TLR5			409 (407)	409 (407)		410 (408)		409	[409]	
TLR5		413								
TLR5			418 (416)							
TLR5	422	422			422	422 (420)		422	422	—
TLR5								424		

TLR5			448 (446)							
TLR5		451								
TLR5		456			455			455	456	—
TLR5			462 (460)							
TLR5	468	468			466	468 (466)		466	468	—
TLR5					471			471		
TLR5			484 (482)							
TLR5			494 (492)							
TLR5			498 (496)				497 (495)			
TLR5					501			501	[501]	
TLR5					502			502	[502]	
TLR5								504		
TLR5								505		
TLR5		508						508	508	—
TLR5	510	510								
TLR5		525	525 (523)						525	—
TLR5		532	**532 (530)**					533	532	—
TLR5		546						548		
TLR5								554		
TLR5					561			561	[561]	
TLR5								562		
TLR5			566 (564)							
TLR5			**569 (567)**	569 (567)				570	[569]	
TLR5			588 (586)							
TLR5			594 (592)			594 (592)	594 (592)		[594]	
TLR5			618 (616)							
TLR5		625					623 (621)		625	—
TLR5			630 (628)				632 (630)			
TLR5			636 (634)							
TLR5	647		646 (644)						647	Membrane region

TLR5		648						
TLR5			652 (650)					
TLR5		659			661 (659)		659	Membrane region
TLR5				676 (674)				
TLR5			682 (680)					
TLR5			692 (690)					
TLR5				723 (721)	722 (720)			
TLR5				744 (742)				
TLR5			849 (847)					
TLR5			856 (854)					
TLR7		3	2				3	Expression guidance site
TLR7				17 (19)				
TLR7	26	26						
TLR7			35 (37)					
TLR7			37 (39)	37 (39)				
TLR7			missing (42)					
TLR7		39	39 (43)				39	Excised region
TLR7			40 (44)					
TLR7		82						
TLR7				85 (89)				
TLR7	99	99		99 (103)			99	Excised region
TLR7			107 (111)	107 (111)			[107]	
TLR7			109 (113)	109 (113)			[109]	
TLR7				121 (125)				
TLR7						124 (128)		
TLR7	144	144						
TLR7				147 (151)				
TLR7	155	155						
TLR7				158 (162)				

TLR7		214 (218)					
TLR7		229 (233)					
TLR7		235 (239)					
TLR7					257		
TLR7	272						
TLR7		278 (283)	278 (283)			[278]	
TLR7					291		
TLR7	298						
TLR7		302 (307)		303 (308)	301		
TLR7					310		
TLR7	313						
TLR7					320		
TLR7					325		
TLR7		336 (341)					
TLR7					345		
TLR7							
TLR7		359 (364)					
TLR7			352 (357)		366		
TLR7			354 (359)				
TLR7			381 (386)				
TLR7	383		383 (388)			383	Excised region
TLR7	386						
TLR7	390						
TLR7			393 (398)		395		
TLR7			408 (413)				
TLR7		416 (421)					
TLR7			420 (425)				
TLR7					427		
TLR7					432		
TLR7	437						

TLR7						444		
TLR7			451 (455)					
TLR7			452 (456)					
TLR7			453 (457)					
TLR7			458 (462)		457 (461)			
TLR7			483 (486)					
TLR7			485 (487)	485 (487)			[485]	
TLR7			488 (490)					
TLR7						490		
TLR7			494 (496)	494 (496)			[494]	
TLR7		509						
TLR7			512 (514)					
TLR7			515 (517)					
TLR7			518 (520)					
TLR7			526 (528)	526 (528)			[526]	
TLR7				528 (530)				
TLR7	537	537						
TLR7			540 (542)					
TLR7		565	564 (566)	564 (566)			565	—
TLR7			595 (597)	597 (599)				
TLR7			635 (637)					
TLR7	638	638						
TLR7		665		665 (667)			665	—
TLR7	669							
TLR7			682 (684)					
TLR7			691 (693)	691 (693)			[691]	
TLR7			694 (696)					
TLR7			695 (697)	695 (697)			[695]	
TLR7		700	698 (700)				700	—
TLR7				717 (719)				

TLR7		732				
TLR7			735 (737)	735 (737)		[735]
TLR7		746				
TLR7	748	748				
TLR7					769 (772)	
TLR7				774 (776)		
TLR7		794				
TLR7			824 (826)			
TLR7			854 (856)			
TLR7				883 (885)		
TLR7		919				
TLR7			942 (944)			
TLR7				1015 (1017)		

Legend: GaGaTLR amino acid position numbering (original numbering of the study given in round brackets)

underlined are result confirmed by several methods independently within the same dataset

in bold are positions of strong evidence

square brackets indicate consensus incongruent with the Galloanserae dataset

consensus in the neighbourhood to Galloanserae selected site (+- 2 aa)

consensus of the Galloanserae selected site with a mammalian study

avian consensus

consensus of the Galloanserae selected site with a mammalian study neighbouring to other selected site detected in birds

References

Alcaide M, Edwards SV (2011) Molecular Evolution of the Toll-Like Receptor Multigene Family in Birds. Molecular Biology and Evolution 28*:*1703-1715

Andersen-Nissen E, Smith KD, Bonneau R, Strong RK, Aderem A (2007) A conserved surface on Toll-like receptor 5 recognizes bacterial flagellin. Journal of Experimental Medicine 204*:*393-403

Areal H, Abrantes J, Esteves PJ (2011) Signatures of positive selection in Toll-like receptor (TLR) genes in mammals. Bmc Evolutionary Biology 11

Atchley WR, Zhao JP, Fernandes AD, Druke T (2005) Solving the protein sequence metric problem. Proceedings of the National Academy of Sciences of the United States of America 102*:*6395-6400

Fornuskova A, Vinkler M, Pages M, Galan M, Jousselin E, Cerqueira F, Morand S, Charbonnel N, Bryja J, Cosson J-F (2013) Contrasted evolutionary histories of two Toll-like receptors (Tlr4 and Tlr7) in wild rodents (MURINAE). Bmc Evolutionary Biology 13

Grueber CE, Wallis GP, Jamieson IG (2014) Episodic positive selection in the evolution of avian toll-like receptor innate immunity genes. PLOS ONE 9*:*e89632

Kim HM, Park BS, Kim JI, Kim SE, Lee J, Oh SC, Enkhbayar P, Matsushima N, Lee H, Yoo OJ, Lee JO (2007) Crystal structure of the TLR4-MD-2 complex with bound endotoxin antagonist eritoran. Cell 130*:*906-917

Ohto U, Fukase K, Miyake K, Shimizu T (2012) Structural basis of species-specific endotoxin sensing by innate immune receptor TLR4/MD-2. Proceedings of the National Academy of Sciences of the United States of America 109*:*7421-7426

Park BS, Song DH, Kim HM, Choi BS, Lee H, Lee JO (2009) The structural basis of lipopolysaccharide recognition by the TLR4-MD-2 complex. Nature 458*:*1191-U130

Philbin VJ, Iqbal M, Boyd Y, Goodchild MJ, Beal RK, Bumstead N, Young J, Smith AL (2005) Identification and characterization of a functional, alternatively spliced Toll-like receptor 7 (TLR7) and genomic disruption of TLR8 in chickens. Immunology 114*:*507-521

Smith SA, Jann OC, Haig D, Russell GC, Werling D, Glass EJ, Emes RD (2012) Adaptive evolution of Toll-like receptor 5 in domesticated mammals. Bmc Evolutionary Biology 12

Vinkler M, Bryjova A, Albrecht T, Bryja J (2009) Identification of the first Toll-like receptor gene in passerine birds: TLR4 orthologue in zebra finch (Taeniopygia guttata). Tissue Antigens 74*:*32-41

Walsh C, Gangloff M, Monie T, Smyth T, Wei B, McKinley TJ, Maskell D, Gay N, Bryant C (2008) Elucidation of the MD-2/TLR4 interface required for signaling by lipid IVa. Journal of Immunology 181*:*1245-1254

Wei TD, Gong J, Jamitzky F, Heckl WM, Stark RW, Rossle SC (2009) Homology modeling of human Toll-like receptors TLR7, 8, and 9 ligand-binding domains. Protein Science 18*:*1684-1691

Wlasiuk G, Khan S, Switzer WM, Nachman MW (2009) A history of recurrent positive selection at the Toll-like receptor 5 in primates. Molecular Biology and Evolution 26*:*937-949

Wlasiuk G, Nachman MW (2010) Adaptation and Constraint at Toll-Like Receptors in Primates. Molecular Biology and Evolution 27*:*2172-2186

Yoon SI, Kurnasov O, Natarajan V, Hong MS, Gudkov AV, Osterman AL, Wilson IA (2012) Structural Basis of TLR5-Flagellin Recognition and Signaling. Science 335*:*859-864
