# Supplementary material for: Protein evolution of Toll-like receptors 4, 5 and 7 within Galloanserae birds
Source: Genet Sel Evol. 2014 Nov 12;46(1):72. doi: 10.1186/s12711-014-0072-6 (PMC4228102; doi:10.1186/s12711-014-0072-6)
Supplement: Additional file 2: Figure S1. — Phylogeny of TLR genes within Galloanserae. Maximum likelihood phylogeny of nucleotide sequences of TLR4 (a), TLR5 (b) and TLR7 (c) in Galloanserae species calculated in PHYML. Figure S2. Phylogenetic relationships between the investigated species. Phylogenetic tree of the investigated species constructed based on consensus avian phylogenetic tool. Figure S3. Secondary structure alignment of Galloanserae TLR. Alignment of secondary structure motifs (α-helices, β-sheets and connecting sequences) in Galloanserae TLR. Figure S4. 3D structural similarity of Galloanserae TLR tertiary structures. Phenograms representing the 3D structural similarity of models of Galloanserae TLR tertiary structures. Figure S5. Species-specific differences in surface electrostatic potential. Visualisation of species-specific differences in surface electrostatic potential on 3D tertiary structure models. [file 12711_2014_72_MOESM2_ESM.pdf]

Title: **Protein evolution of Toll-like receptors 4, 5 and 7 within Galloanserae birds**
Authors: Michal Vinkler, Hana Bainová & Josef Bryja
Document: Additional file 2

Figure S1 - Phylogenetic relationships between the investigated species constructed based on consensus avian phylogenetic tool available at http://birdtree.org/ (Jetz et al. 2012).

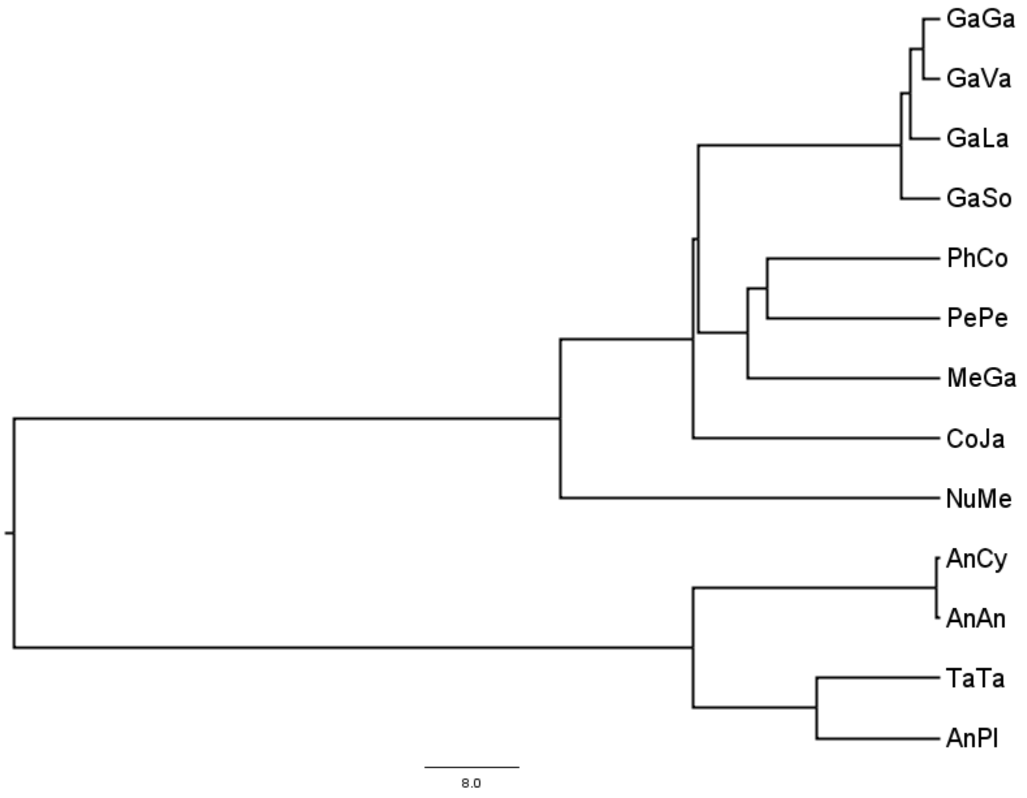

Figure S2 - Phylogeny of *TLR* genes within Galloanserae.

Maximum likelihood phylogeny of nucleotide sequences of *TLR4* (a), *TLR5* (b) and *TLR7* (c) in Galloanserae species calculated in PHYML online software. Orthologous sequences of human (HoSa) and mouse (MuMu) were used as outgroups. Small numbers indicate the bootstrap values (only those higher than 75 are shown).

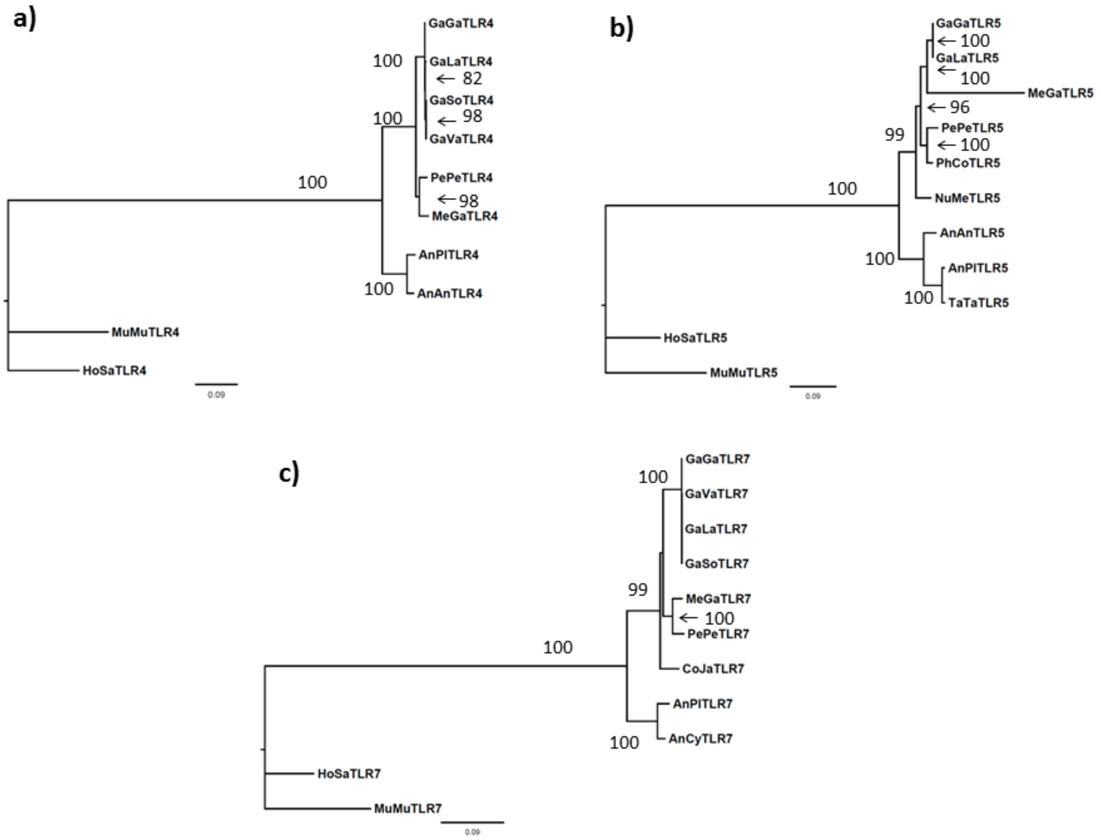

Figure S3 - Secondary structure alignment of Galloanserae TLRs.

(a) TLR4, (b) TLR5 and (c) TLR7: H = α-helix, S = β-sheet, C = connecting sequence, confidence score provided in third line.

a)

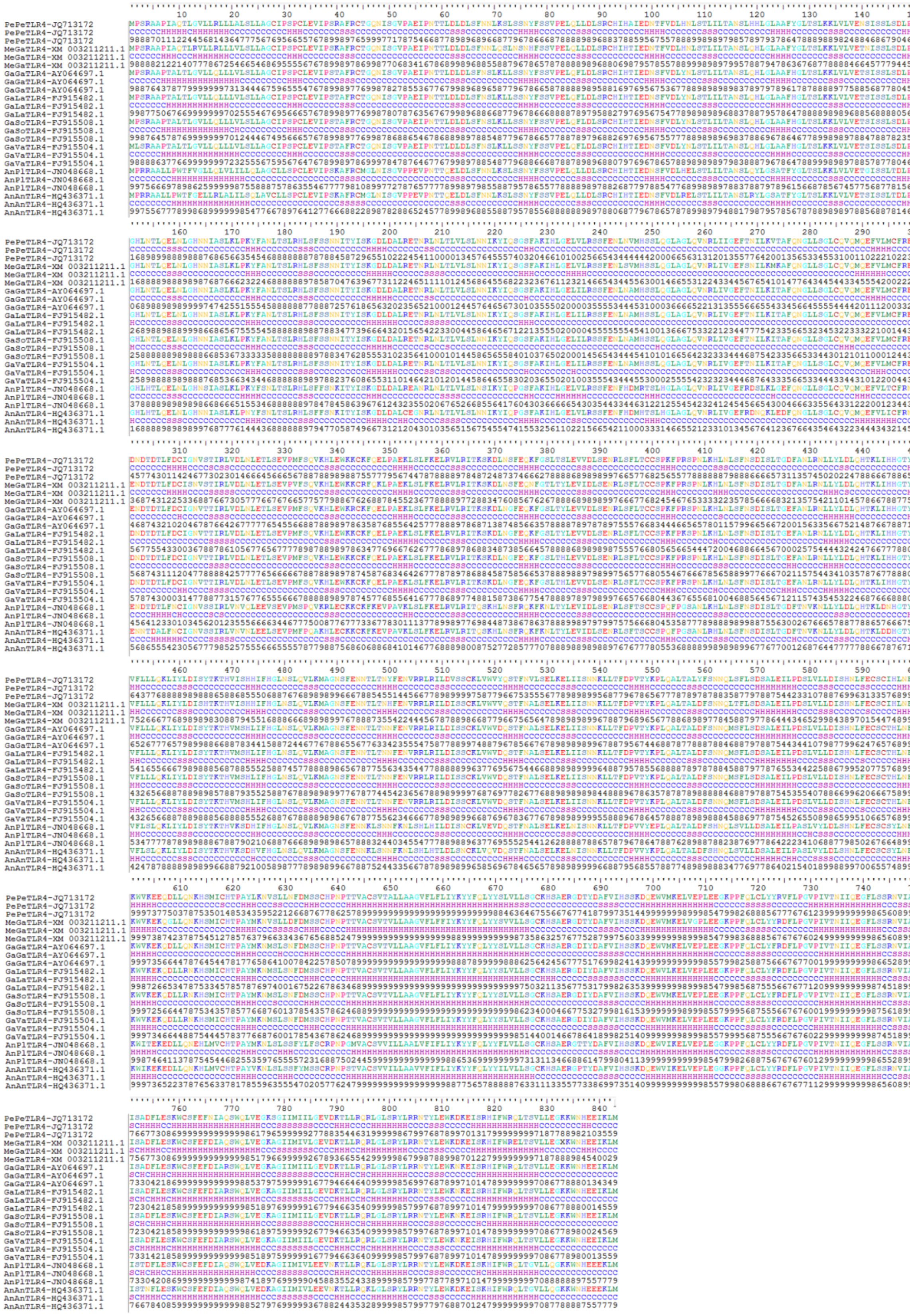

b)

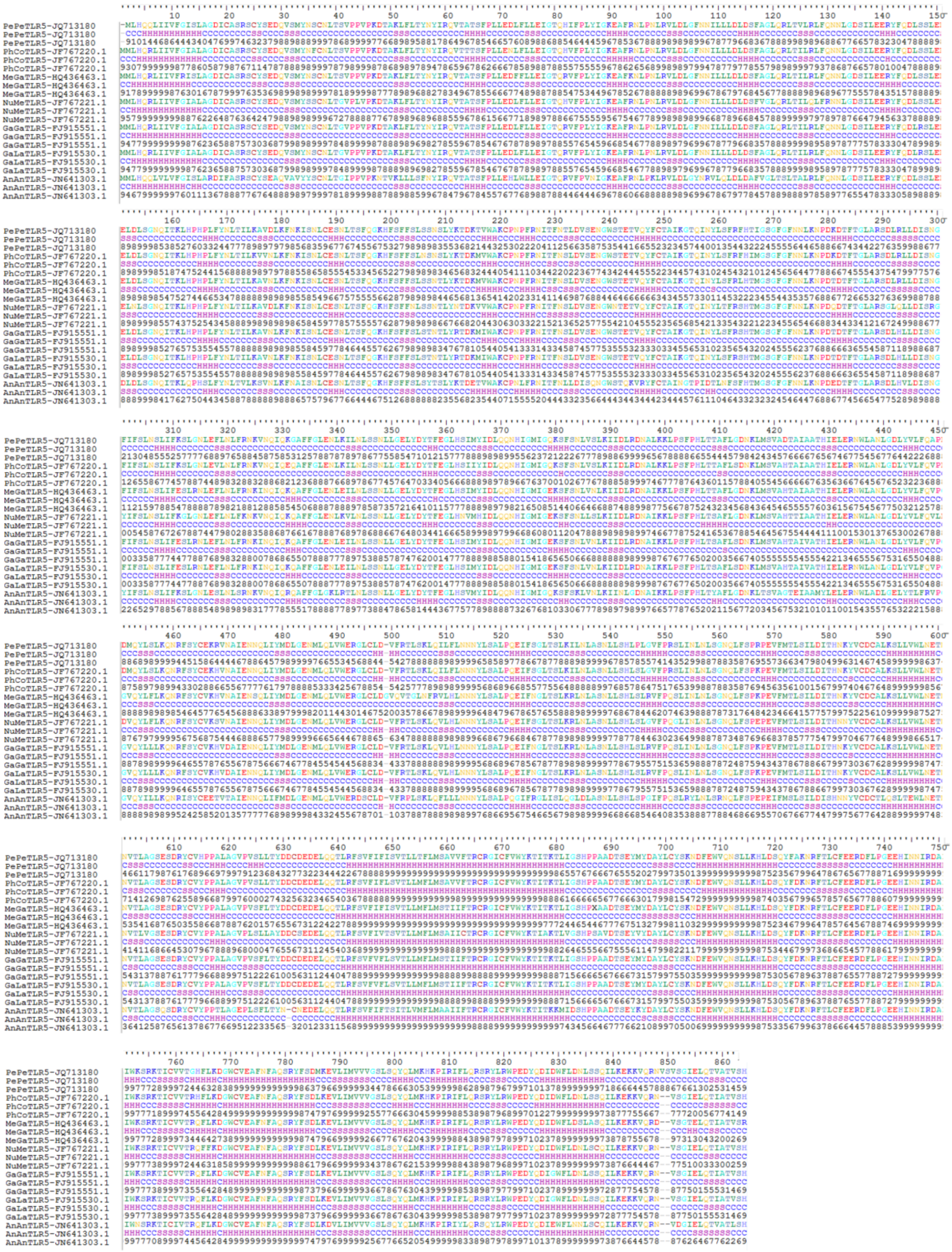

c)

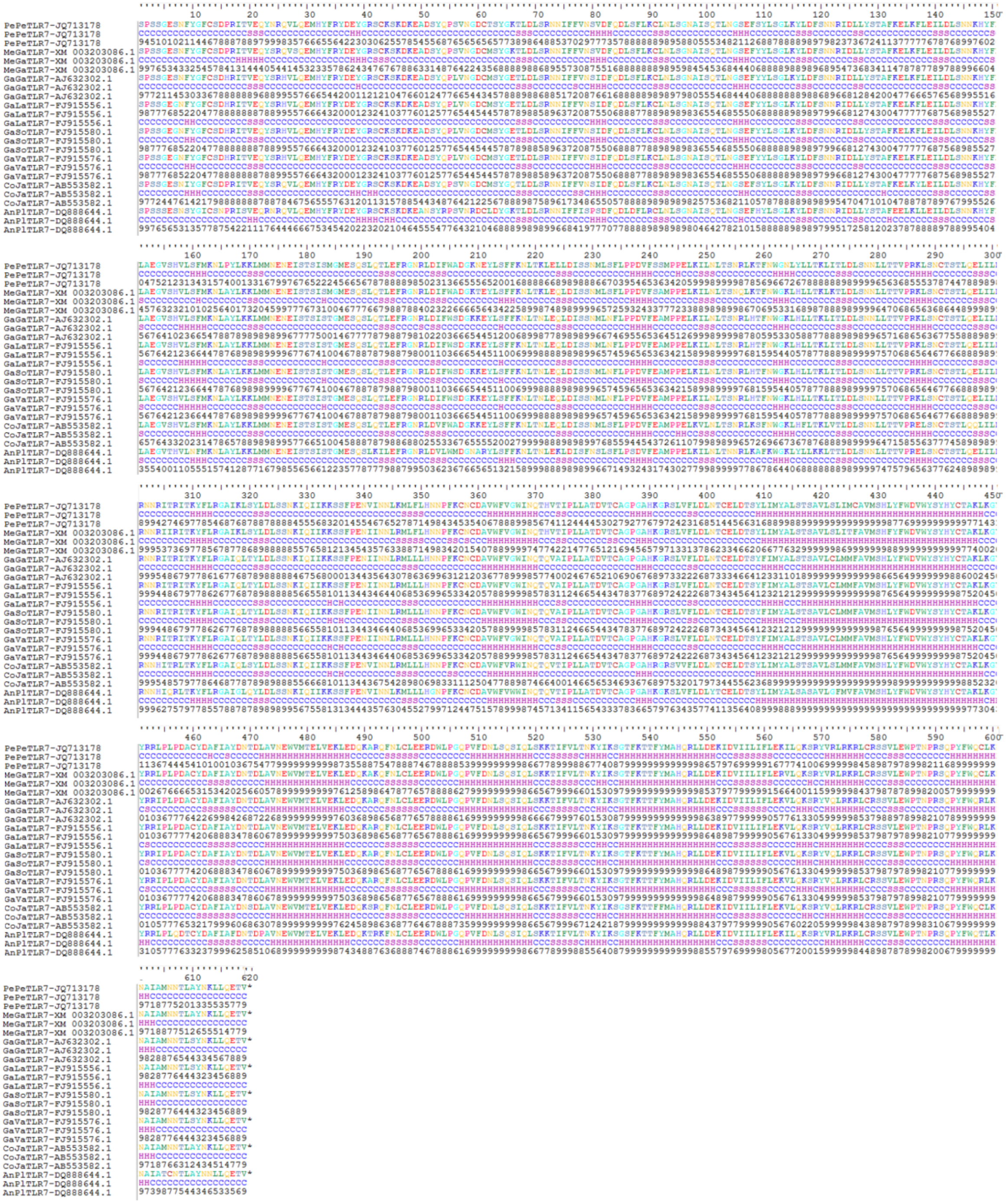

Figure S4 - Phenograms representing the 3D structural similarity of models of Galloanserae TLRs tertiary structures.

a) TLR4 ECD b) TLR5 ECD c) TLR7 ECD d) TLR4 TIR domain e) TLR5 TIR domain f) TLR7 TIR domain. X axis shows RMSDs of compared modelled structures in Å.

a)

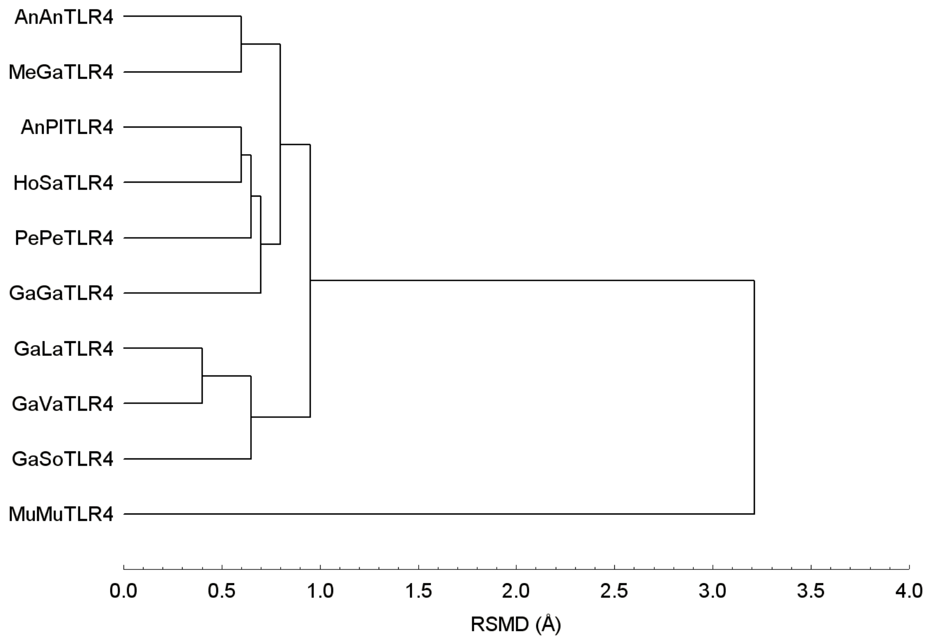

b)

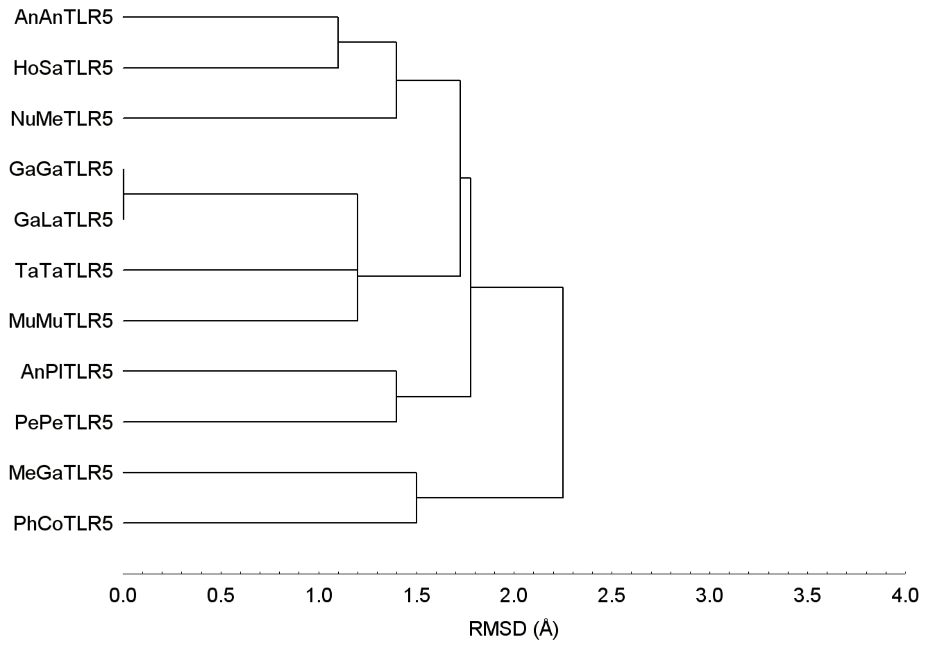

c)

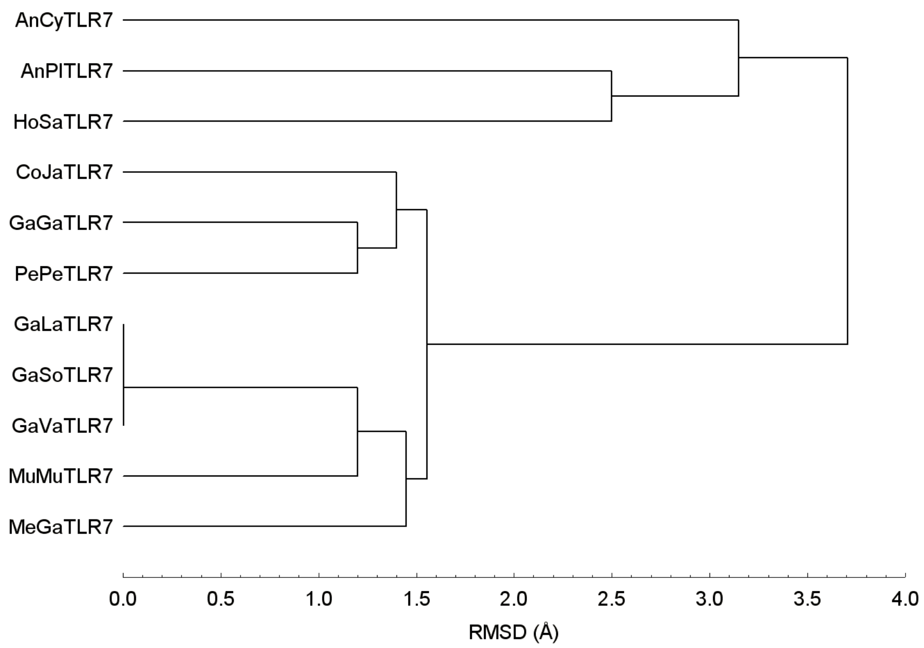
AnCyTLR7
AnPlTLR7
HoSaTLR7
CoJaTLR7
GaGaTLR7
PePeTLR7
GaLaTLR7
GaSoTLR7
GaVaTLR7
MuMuTLR7
MeGaTLR7
0.0
0.5
1.0
1.5
2.0
2.5
3.0
3.5
4.0
RMSD (Å)

d)

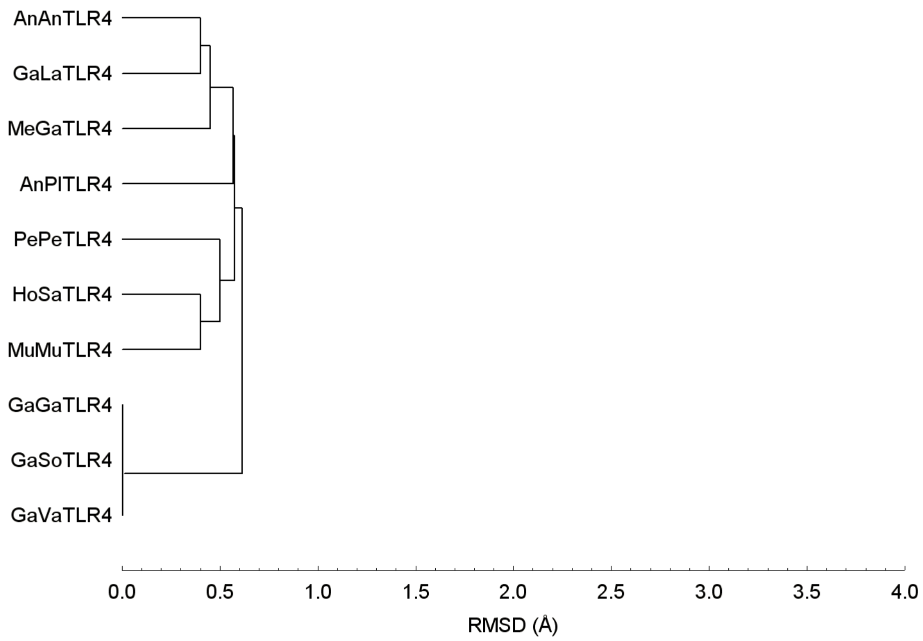
AnAnTLR4
GaLaTLR4
MeGaTLR4
AnPlTLR4
PePeTLR4
HoSaTLR4
MuMuTLR4
GaGaTLR4
GaSoTLR4
GaVaTLR4
0.0
0.5
1.0
1.5
2.0
2.5
3.0
3.5
4.0
RMSD (Å)

e)

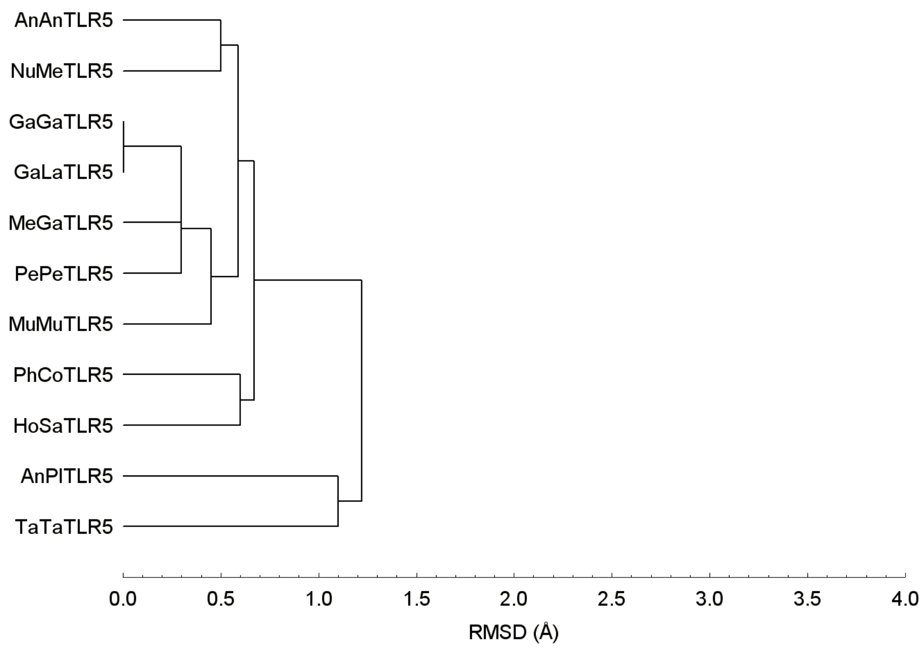
AnAnTLR5
NuMeTLR5
GaGaTLR5
GaLaTLR5
MeGaTLR5
PePeTLR5
MuMuTLR5
PhCoTLR5
HoSaTLR5
AnPlTLR5
TaTaTLR5
0.0
0.5
1.0
1.5
2.0
2.5
3.0
3.5
4.0
RMSD (Å)

f)

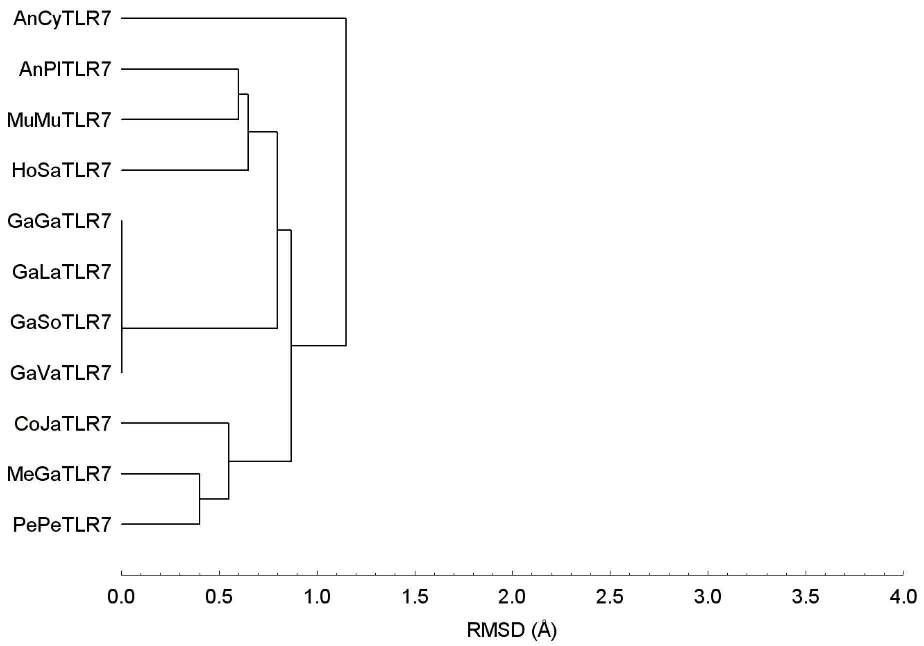
AnCyTLR7
AnPlTLR7
MuMuTLR7
HoSaTLR7
GaGaTLR7
GaLaTLR7
GaSoTLR7
GaVaTLR7
CoJaTLR7
MeGaTLR7
PePeTLR7
0.0
0.5
1.0
1.5
2.0
2.5
3.0
3.5
4.0
RMSD (Å)

Figure S5 - Visualisation of species-specific differences in surface electrostatic potential. a) TLR4 ECD, b) TLR5 ECD, c) TLR7 ECD, d) TLR4 TIR, e) TLR5 TIR, f) TLR7 TIR. Positive surface charge is highlighted in red, negative charge in blue. In ECDs the predicted ligand-binding sites (Andersen-Nissen et al. 2007; Kim et al. 2007; Park et al. 2009; Walsh et al. 2008; Wei et al. 2009; Yoon et al. 2012) are highlighted by yellow lining in the GaGaTLR models. Given the high similarity of the structures belonging to individual *Gallus* species and similarity between AnPlTLR5 and TaTaTLR5, only GaGaTLRs and AnPlTLR5 are shown.

a)

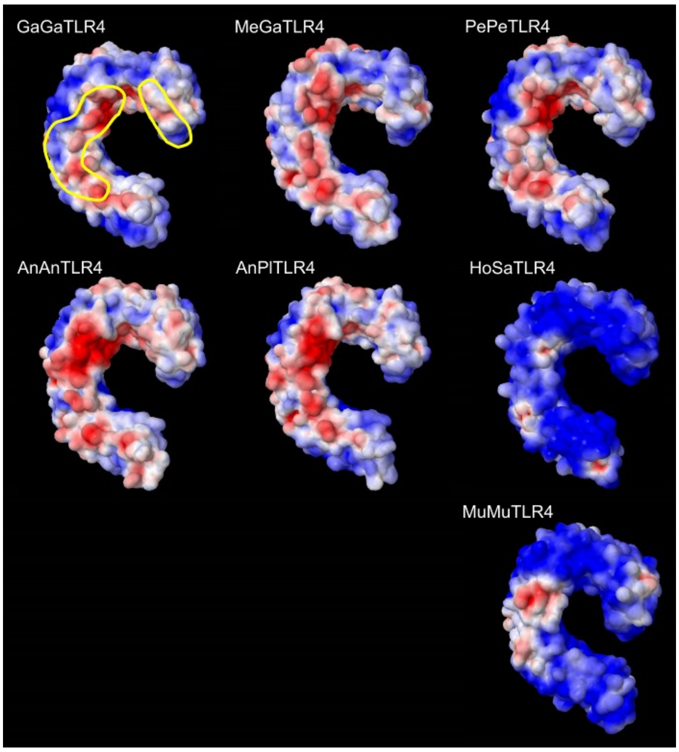

b)

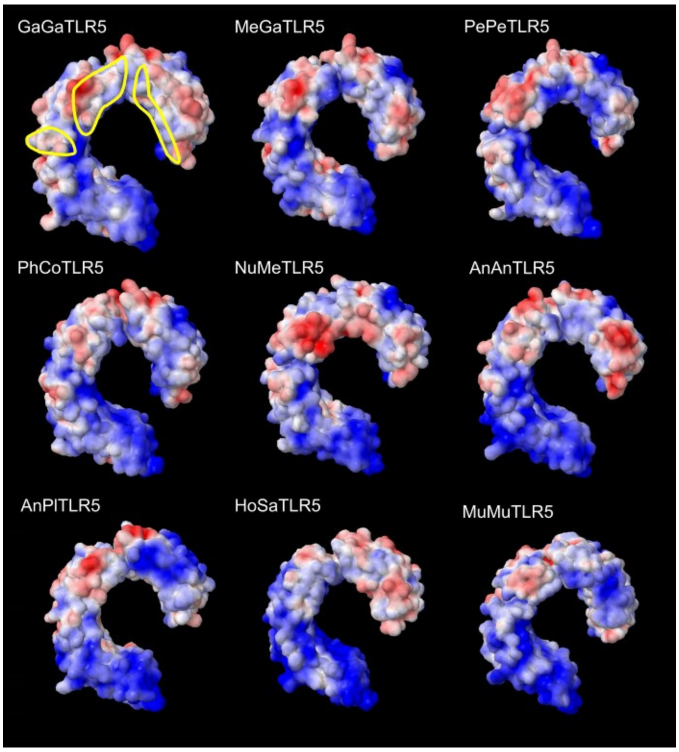

c)

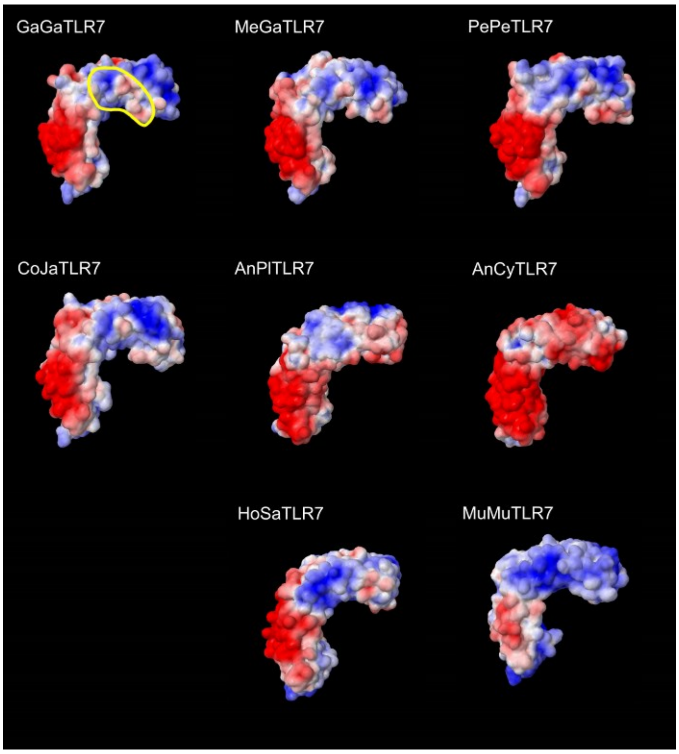
GaGaTLR7
MeGaTLR7
PePeTLR7
CoJaTLR7
AnPlTLR7
AnCyTLR7
HoSaTLR7
MuMuTLR7

d)

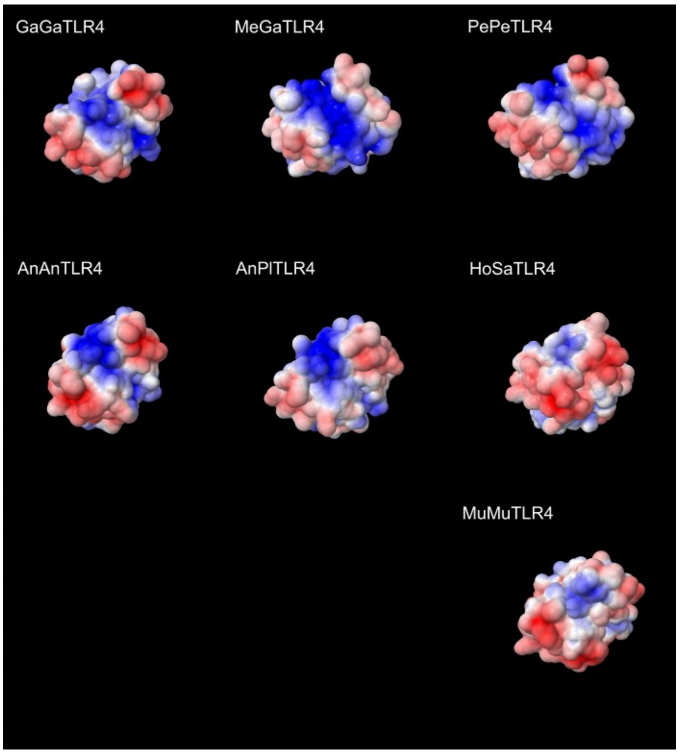
GaGaTLR4
MeGaTLR4
PePeTLR4
AnAnTLR4
AnPlTLR4
HoSaTLR4
MuMuTLR4

e)

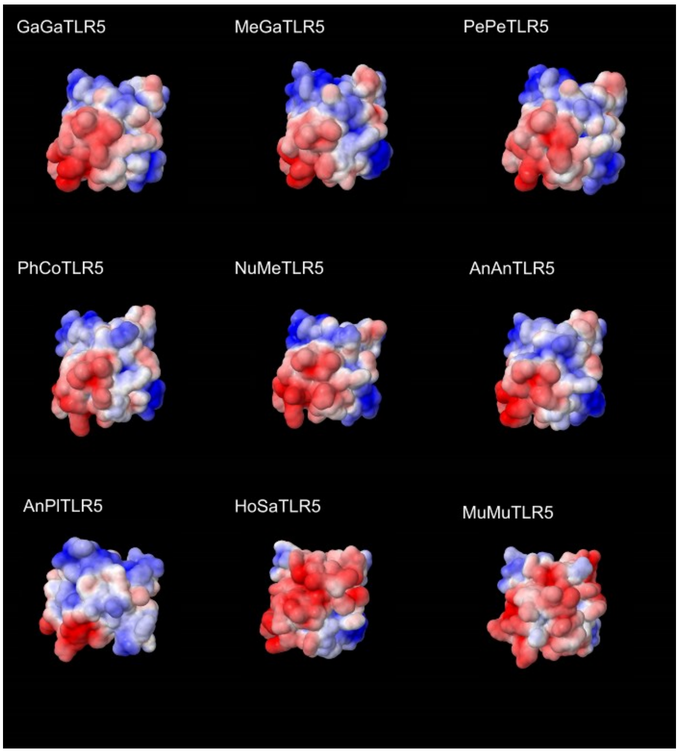
GaGaTLR5
MeGaTLR5
PePeTLR5
PhCoTLR5
NuMeTLR5
AnAnTLR5
AnPlTLR5
HoSaTLR5
MuMuTLR5

f)

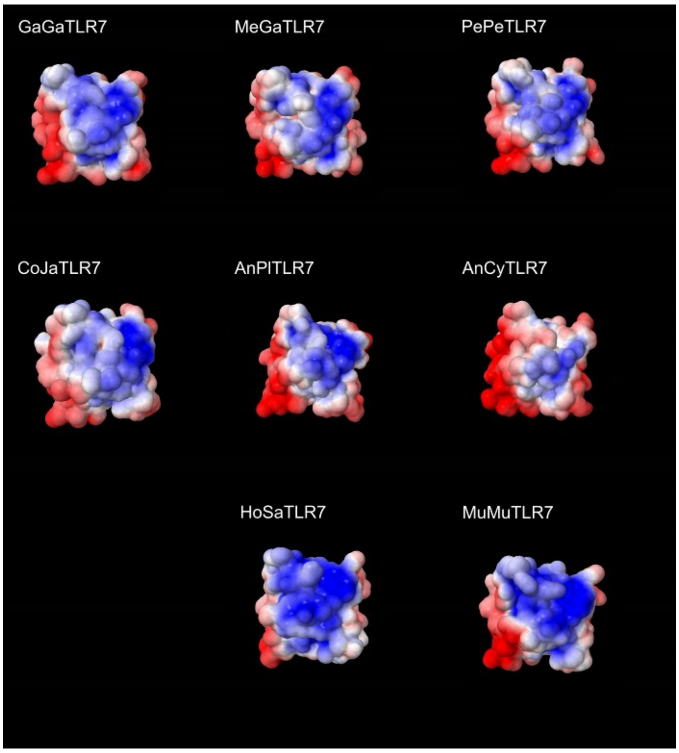
GaGaTLR7
MeGaTLR7
PePeTLR7
CoJaTLR7
AnPlTLR7
AnCyTLR7
HoSaTLR7
MuMuTLR7

References

Andersen-Nissen E, Smith KD, Bonneau R, Strong RK, Aderem A (2007) A conserved surface on Toll-like receptor 5 recognizes bacterial flagellin. Journal of Experimental Medicine 204*:*393-403

Jetz W, Thomas GH, Joy JB, Hartmann K, Mooers AO (2012) The global diversity of birds in space and time. Nature 491*:*444-448

Kim HM, Park BS, Kim JI, Kim SE, Lee J, Oh SC, Enkhbayar P, Matsushima N, Lee H, Yoo OJ, Lee JO (2007) Crystal structure of the TLR4-MD-2 complex with bound endotoxin antagonist eritoran. Cell 130*:*906-917

Park BS, Song DH, Kim HM, Choi BS, Lee H, Lee JO (2009) The structural basis of lipopolysaccharide recognition by the TLR4-MD-2 complex. Nature 458*:*1191-U130

Walsh C, Gangloff M, Monie T, Smyth T, Wei B, McKinley TJ, Maskell D, Gay N, Bryant C (2008) Elucidation of the MD-2/TLR4 interface required for signaling by lipid IVa. Journal of Immunology 181*:*1245-1254

Wei TD, Gong J, Jamitzky F, Heckl WM, Stark RW, Rossle SC (2009) Homology modeling of human Toll-like receptors TLR7, 8, and 9 ligand-binding domains. Protein Science 18*:*1684-1691

Yoon SI, Kurnasov O, Natarajan V, Hong MS, Gudkov AV, Osterman AL, Wilson IA (2012) Structural Basis of TLR5-Flagellin Recognition and Signaling. Science 335*:*859-864
