# Supplementary material for: Protein evolution of Toll-like receptors 4, 5 and 7 within Galloanserae birds
Source: Genet Sel Evol. 2014 Nov 12;46(1):72. doi: 10.1186/s12711-014-0072-6 (PMC4228102; doi:10.1186/s12711-014-0072-6)
Supplement: Additional file 3: Section S1. — Variability in Galloanserae TLR basic protein features. Description of basic protein features in Galloanserae TLR. Section S2. Evolution of TLR secondary structures within the Galloanserae lineage. Description of secondary structure similarities in Galloanserae TLR. Section S3. Variability in features of predicted ligand-binding residues. Detailed information on amino acid binding features at the functional sites known in other vertebrates. Section S4. Analysis of positive selection. Detailed information and discussion on the results of the positive selection analysis. [file 12711_2014_72_MOESM3_ESM.pdf]

Title: **Protein evolution of Toll-like receptors 4, 5 and 7 within Galloanserae birds**

Authors: Michal Vinkler, Hana Bainová & Josef Bryja

Document: Additional file 3

Section S1 Variability in Galloanserae TLR basic protein features

All examined TLR proteins showed typical TLR structure composed of an initial signal peptide, followed by extracellular LRRs, a transmembrane region and a TIR domain. The signal peptide extend was uniform in its length in TLR4 and TLR7 in all included species (30 aa and 25 aa, respectively) but it differed in size among Galloanserae species in TLR5 with length ranging from 16 aa to 21 aa [See Additional file 1, Table S3]. Also the number of predicted LRRs remarkably varied among orthologous TLRs [See Additional file 1, Table S3]. Both N-terminal and C-terminal caps of the most external LRRs (forming LRRNT and LRRCT, respectively) were present only in TLR7, while in TLR4 and TLR5 only the C-terminal caps were predicted, uniformly in all investigated species.

Section S2 Evolution of TLR secondary structures within the Galloanserae lineage

The secondary structure variability detected in the present study in the Galloanserae TLRs was low. The ECDs did not differ from the intracellular regions in the level of variability of the predicted secondary structures. Nevertheless, regions between residues 225-311, 412-438 and 628-636 in TLR4 and 407-431 and 623-626 in TLR5 were variable among individual species. TLR7 was the most conservative of the TLRs investigated. Although the predicted variability seems only minor, in TLR4 the first two regions comprise sites known in mammals to be involved in MD-2 binding, TLR4–TLR4 dimerization, and even LPS binding (Ohto et al. 2012). These results, thus, suggest that some of the protein conformation variability within the Galloanserae clade could be associated with potential differences in the mode of ligand binding.

Section S3 Variability in features of predicted ligand-binding residues

In TLR4 the amino acid binding features at the functional sites known in mammals remained probably unchanged in 19 out of 29 sites (66%). Similarly, in TLR5 32 out of 56 (57%) functional sites remained physiochemically similar. The difference between zebrafish functional sites identified by Yoon et al. (2012) and their avian counterparts is dramatic (physiochemical similarity only in 23 out of 45) and further increased by missing amino acids. This indicates that flagellin binding is probably not completely identical in fish and amniotes. Nonetheless, if we do not include sites that were recognised only in zebrafish and

focus only on those predicted by Andersen-Nissen et al. (2007) for mammals, then 10 out of the 11 (91%) functional sites are conservative. Very similar to this is the situation in TLR7 where 7 out of 9 functional sites show physiochemical conservatism. Little (TLR5) or no (TLR7) interspecific polymorphism in the mammal-predicted ligand-binding residues within the Galloanserae lineage, however, suggests that the ligand-binding properties in both these proteins may be reasonably conserved between birds and mammals. Intriguingly, in TLR4 the residues 449 (= HoSaTLR4-F440) and 472 (= HoSaTLR4-F463) that have been previously reported as key for LPS binding (Resman et al. 2009) are altered in birds when compared to mammals. At all these sites nonpolar F residues are required in humans (Resman et al. 2009). In contrast, in Galloanserae birds there is uniformly aromatic hydrophilic Y at site 449 and tiny hydrophilic S at site 472. This means that either the model proposed by Resman et al. (2009) is incorrect or, more likely, that there is a substantial difference between birds and mammals in TLR4-MD-2-LPS binding. Moreover, there are several sites in TLR4 (268, 397, 428, 445 and 454) at which Galliformes systematically differ from Anserifomes. Most of these residues (268, 397 and 445) were found in humans to be involved in interaction with LPS (Park et al. 2009). This suggests differences in LPS binding between Galliformes and Anseriformes. Again, we found only little interspecific variability in the predicted key binding residues within the two individual avian orders. Only limited polymorphism has been revealed on two well-known human functional SNP sites (Arbour et al. 2000; Rallabhandi et al. 2006). At the site of avian 303 which is identical to human D299G SNP position aspartic acid (D) was invariantly present (except for AnAnTLR4-N303). Residues on position 408 (equivalent to HoSaTLR4-T399I SNP) were variable, with proline being present in Galliform birds and alanine being present in Anseriform birds.

Section S4 Analysis of positive selection

Except for one position that was detected in the transmembrane domain (TMD) of TLR5, all positively selected sites detected in Galloanserae (19) were located in the extracellular domains (ECDs) [See Additional file 1, Tables S7 and S8]. Only one positively selected site (26 in TLR7) was present in the signal peptide, possibly influencing the TLR7 expression into membranes. Out of the 18 remaining positively selected sites identified in this study only five (TLR4: 343; TLR5: 180, 209, 342, 379) were located either precisely in or in close proximity to any of the predicted functionally important sites [See Additional file 1, Table S5]. Residues 343 in TLR4 and 180 and 379 in TLR5 seem to be potentially more evolutionarily relevant since the substitutions on these sites significantly alter amino acid properties and may, hence, affect ligand binding.

The ConSurf analysis [See Additional file 1, Table S9] identified 26, 28 and 25 reliably non-conservative sites in TLR4, TLR5 and TLR7, respectively. Out of these, only one site (TLR7) belonged to the TIR domain, three (TLR4: 1, TLR5: 2) to TMD and two were located in the intracellular domain of TLR4 outside of the TIR domain. The functional effect of the substitutions on the TLR7 TIR position 919 is difficult to assess but the impact of the substitutions in the TMDs seem to be only unimportant. Six, one and one positions, respectively for TLR4, TLR5 and TLR7, belonged to signal peptide regions with possible effects on protein expression. In TLR4 substitutions at four sites (40, 268, 270 and 471) might affect TLR4-MD-2 dimerization. Substitutions at three sites could influence LPS binding by TLR4 (268, 444 and 471). Finally, substitutions at two sites could affect TLR4-TLR4 dimerization (444 and 512). Except for the substitutions at the position 512 are the alternations at the functional positions physiochemically non-conservative and might, therefore, influence LPS binding. In TLR5 substitutions at positions 82, 183, 207, 209, 216, 264 and 342 might affect flagellin binding. At least some of the substitutions at the positions 183, 207, 209, 216, 264 and 342 might alter the features of the flagellin-binding residues. In TLR7 there was no site with potentially functional effect identified by the analysis.

Intriguingly, several sites in each receptor were identified as positively selected by several evolutionary studies conducted either in birds or in mammals [See Additional file 1, Table S10]. In TLR4 sites 205, 273, 274, 275 and 301 were detected in Galloanserae birds and mammals and sites 246, 270, 274, 301 and 406 were detected both in Galloanserae and other birds. Moreover, sites 343, 444, 471, 512, 521, 627 and 686 identified in the present study were in close proximity to some other positively selected sites identified in other taxa. Out of these, sites 270 and 471 might influence TLR-4-MD-2 dimerization, positions 343, 444 and 471 LPS-binding and site 512 TLR4-TLR4 dimerization. In TLR5 sites 183, 209, 264, 314, 342, 422, 525 and 532 were detected in Galloanserae birds and mammals and sites 209, 264, 281, 293, 422 and 508 were identified both in Galloanserae and other birds. Furthermore, sites 216, 259, 379, 456, 468, 625, 647 and 659 identified in the present study were in close proximity to some other positively selected sites identified in other taxa. Out of these, sites 183, 209, 216, 264, 342 and 379 might influence the structure of the flagellin-binding interface. In TLR7 sites 39, 99, 383 and 665 were detected in Galloanserae birds and mammals, no sites were consistently identified in Galloanserae and other birds and sites 3, 565 and 700 that were identified in the present study were located in close proximity to some

other positively selected sites identified in other taxa. Out of these, sites none was located in the predicted ligand-binding region.

Based on the evidence summarised above we may propose several sites that should be investigated in closer detail. Mainly the sites 270, 343, 444 and 471 in TLR4 and 180, 183, 209, 216, 264, 342 and 379 in TLR5 are clear key candidates for further research on functional significance of selection acting on TLRs in birds. Since no selection on this site has been evidenced in mammals or other birds, we may hypothesise that the site 268 in TLR4 may be of some special importance in the evolution of Galloanserae birds. Although we do not know the functional importance of the sites, based solely on congruence of the selection analyses in birds and mammals special attention should be also paid to positions 244-246, 273-275, 301 in TLR4 and 293-294, 314, 342, 422, 525,532-533 in TLR5.

References

Andersen-Nissen E, Smith KD, Bonneau R, Strong RK, Aderem A (2007) A conserved surface on Toll-like receptor 5 recognizes bacterial flagellin. Journal of Experimental Medicine 204*:*393-403

Arbour NC, Lorenz E, Schutte BC, Zabner J, Kline JN, Jones M, Frees K, Watt JL, Schwartz DA (2000) TLR4 mutations are associated with endotoxin hyporesponsiveness in humans. Nature Genetics 25*:*187-191

Ohto U, Fukase K, Miyake K, Shimizu T (2012) Structural basis of species-specific endotoxin sensing by innate immune receptor TLR4/MD-2. Proceedings of the National Academy of Sciences of the United States of America 109*:*7421-7426

Park BS, Song DH, Kim HM, Choi BS, Lee H, Lee JO (2009) The structural basis of lipopolysaccharide recognition by the TLR4-MD-2 complex. Nature 458*:*1191-U130

Rallabhandi P, Bell J, Boukhvalova MS, Medvedev A, Lorenz E, Arditi M, Hemming VG, Blanco JCG, Segal DM, Vogel SN (2006) Analysis of TLR4 polymorphic variants: New insights into TLR4/MD-2/CD14 stoichiometry, structure, and signaling. Journal of Immunology 177*:*322-332

Resman N, Vasl J, Oblak A, Pristovsek P, Gioannini TL, Weiss JP, Jerala R (2009) Essential Roles of Hydrophobic Residues in Both MD-2 and Toll-like Receptor 4 in Activation by Endotoxin. Journal of Biological Chemistry 284*:*15052-15060

Yoon SI, Kurnasov O, Natarajan V, Hong MS, Gudkov AV, Osterman AL, Wilson IA (2012) Structural Basis of TLR5-Flagellin Recognition and Signaling. Science 335*:*859-864
